# High‐Speed Interferometric Scattering Tracking Microscopy of Compartmentalized Lipid Diffusion in Living Cells

**DOI:** 10.1002/cphc.202400407

**Published:** 2025-09-28

**Authors:** Francesco Reina, Christian Eggeling, Christoffer Lagerholm

**Affiliations:** ^1^ Institute of Applied Optics and Biophysics Friedrich Schiller University Jena 07743 Jena Germany; ^2^ Department of Biophysical Imaging Leibniz‐Institute for Photonic Technologies e.V. 07745 Jena Germany; ^3^ Leibniz Centre for Photonics in Infection Research (LPI) 07745 Jena Germany; ^4^ MRC Human Immunology Unit and Wolfson Imaging Centre MRC Weatherall Institute of Molecular Medicine John Radcliffe Hospital University of Oxford Oxford OX3 9DS UK; ^5^ Max Perutz Labs University of Vienna 1030 Vienna Austria; ^6^ Jena Center for Soft Matter (JCSM) 07745 Jena Germany; ^7^ Cell imaging and Cytometry (CIC) Core Turku Bioscience University of Turku and Åbo Akademi University FI‐20520 Turku Finland; ^8^ Oxford ZEISS Centre of Excellence Kennedy Institute of Rheumatology University of Oxford Oxford OX3 7FY UK

**Keywords:** localization precision, nanoparticles, phospholipids, plasma membranes, single particle tracking localization precision

## Abstract

Lateral diffusion measurements have been ‐used to infer information about the nano‐organization of membranes. We employed interferometric scattering (ISCAT) microscopy at an acquisition rate of 2 kHz to revisit the diffusion dynamics of a phospholipid analog on the plasma membrane of Ptk2 cells. The ISCAT trajectory data are analyzed with an unbiased, statistics‐driven pipeline to identify the most likely diffusion mode from a set of plausible diffusion modes. At the ensemble average level, the data are best described as transient compartmentalized diffusion with an average compartment size of 100–110 nm, transient confinement time of 8–10 ms, intracompartmental diffusion coefficient of 0.7–0.9 μm^2^ s^−1^, and intercompartmental diffusion coefficient of 0.3–0.4 μm^2^ s^−1^. The same analysis applied at the single‐trajectory level identifies a complex variety of diffusion modes with 7–8% free, 13–14% confined, 40% transient compartmentalized, and 40% anomalous diffusion. Measurements with larger (Ø40 nm) as compared to smaller (Ø20 nm) gold nanoparticles are found to influence the diffusion rate and confinement strength, but not the underlying lipid diffusion modes. Using Monte Carlo simulations, these experimental results are explored in the wider context of relevant literature. This analysis paints a unifying picture of lipid diffusion on mammalian cell membranes transcending differences between experimental techniques.

## Graphical abstract

Scheme for the labeling of the cell membrane. The scattering tags, gold nanoparticles of two different diameters (Ø20 nm and Ø40 nm), target the biotinylated lipids inserted in the cellular membrane, owing to their streptavidin coating. The possible, although not certain, effects of cross‐linking are also highlighted (dashed line).

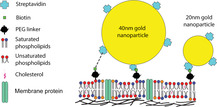

## 1 Introduction

The detection of “hop” diffusion on the outer leaflet of the cellular plasma membrane is a controversial topic in modern cellular biophysics. This diffusive behavior is induced by the presence of compartments or corrals on the plasma membrane, which cause a faster short‐range diffusion within the compartments, and slower long‐range diffusion between compartments. This behavior has been observed through high‐speed single particle tracking (SPT),^[^ ^1^ ^]^ single fluorescent‐molecule imaging (SFMI),^[^ ^2^ ^]^ and super‐resolution stimulated emission depletion (STED) fluorescence correlation spectroscopy (STED‐FCS) microscopy.^[^ ^3^ ^]^ The transition between the two diffusion modes has been observed in the time‐domain of <10 ms, and ≈50–200 nm in the space‐domain. This diffusive behavior is compatible with e.g., the picket‐fence model of the cellular plasma membrane, where the cortical F‐actin meshwork in interplay with linked picket‐like transmembrane structures induces the compartmentalization,^[^ ^4^ ^]^ the role of transmembrane proteins, such as CD44, in affixing the actin cytoskeleton to the plasma membrane,^[^ ^5^ ^]^ as well as alternative potential mechanisms such as the inherent 3D topology of the cellular membranes.^[^ ^6^ ^]^

Initial observations of transient compartmentalized “hop” diffusion suggested that the confinement strength of phospholipids in the outer leaflet of the plasma membrane was very strong. This is highlighted by the confinement strength metric *S*_conf_, defined by Wieser et al.^[^ ^7^ ^]^ as the ratio of the intracompartmental diffusion coefficient *D*_μ_ and the intercompartmental diffusion coefficient *D*_M_, *S*_Conf_ = *D*_μ_/*D*_M_. Initially, values of this metric were reported as high as *S*_Conf_ ≈ 30 for lipids labeled with Ø40 nm gold (Au) nanoparticles (NPs) and imaged at an acquisition rate of 40 kHz.^[1a]^ This strong confinement would presumably require, e.g., very densely packed membrane protein pickets or highly variable topological features, neither of which has been directly observed. More recent observations, including our measurements with the complementary STED‐FCS technique, with a minimally invasive Atto647N dye‐labeled saturated phospholipid analog, and high‐speed SFMI, with a minimally invasive Cy3 dye‐labeled unsaturated phospholipid analog, have confirmed the presence of transient compartmentalized “hop” diffusion.^[^ ^3^ ^]^ Significantly, these studies indicated that the confinement strength was much weaker, with STED‐FCS studies suggesting *S*_Conf_ ≈ 0.6 μm^2^ s^−1^/0.3 μm^2^ s^−1^ = 2 in NRK cells, and *S*_Conf_ ≈ 0.7 μm^2^ s^−1^/0.4 μm^2^ s^−1^ ≈ 1.8 in IA32 MEFs,^[^ ^3^ ^]^ and a very recent SMFI study suggesting *S*_Conf_ ≈ 0.83 μm^2^ s^−1^/0.30 μm^2^ s^−1^ ≈ 2.8 in human epithelial T24 cells.^[^ ^2^ ^]^ These more recent studies suggest a consistent compartment size of about 100 nm, and a transient confinement time of about 10 ms. This much weaker confinement strength, which primarily originated from much lower values of the intracompartmental diffusion coefficient *D*_μ_ could explain why no extreme barriers (e.g., in form of the aforementioned densely packed pickets) have thus far been directly observed. The latter much weaker confinement strength could also explain why transient compartmentalized diffusion was not observed in a related SFMI study of the lipid‐anchored protein CD59 that had been post‐labeled with a minimal invasive Alexa Fluor 647 labeled Fab antibody fragment in the plasma membrane of live T24 cells,^[^ ^7^ ^]^ or by the initial STED‐FCS study in the plasma membrane of live Ptk2 cells.^[^ ^8^ ^]^

One of the challenges with investigating transient compartmentalized (“hop”) diffusion is that relevant experimental methods require both high spatial resolution as compartment sizes are usually below 200 nm in size, and high temporal resolution for distinguishing intra‐ from inter‐compartmental dynamics. These spatiotemporal regimes are only accessible to few experimental techniques, amongst which optical microscopy has proven particularly successful, with approaches such as super‐resolution STED‐FCS techniques.[^1^, ^2^, ^8^, ^9^] A particular strength of single‐molecule tracking techniques such as SPT and SFMI over STED‐FCS is the ability to explore heterogeneity among single‐molecule behaviors. Yet this strength is difficult to fully exploit due to the faster image acquisition rate required to observe hop diffusion^[^ ^8^ ^]^ and because long observations are generally required to obtain adequate levels of detail. Due to single fluorophore photobleaching classic SMFI often only record very short trajectories, and consequently the required precision is usually achieved by ensemble average analysis of all single‐molecule trajectories, which precluded a detailed analysis of the heterogeneity amongst single‐molecule trajectories. Single‐molecule trajectory analysis of compartmentalized “hop” diffusion has consequently thus far rather been limited to SPT studies with larger, typically Ø40 nm Au NPs (e.g., ref. [1a] and later related work by the Kusumi lab), and quantum dots.^[1d]^

There are, however, a number of recent approaches that address these experimental limitations. These include, for example, recent developments with reversible dye‐labeling technology, such as DNA paint, that circumvent the photo‐bleaching issue and thereby provide attractive alternatives for future studies.^[^ ^10^ ^]^ High‐speed tracking single‐molecule tracking with MINFLUX microscopy with minimally invasive single dye‐labeled molecules of interest has also emerged as promising technique. It has, for example, been extensively validated for tracking directional motion of kinesin along microtubules with nanometer precision at millisecond temporal resolution,^[^ ^11^ ^]^ and has been showcased for tracking the diffusion of single lipid analogs and membrane proteins in model and live cell membranes.^[^ ^12^ ^]^ A recent detailed treatise of the accuracy of MINFLUX tracking is also likely to help move this forward in the near future.^[^ ^13^ ^]^ There are also a number of recent implementations of deep learning approaches that are making great strides in improving the accuracy of analysis of short single‐molecule trajectories, e.g.^[^ ^14^ ^]^

In this study, we have employed a custom‐built interferometric scattering microscopy (ISCAT) setup^[^ ^15^ ^]^ to revisit the diffusion dynamics of phospholipids in the plasma membrane of live Ptk2 cells at physiological conditions. ISCAT microscopy is a relatively recent evolution of interference reflection microscopy^[^ ^16^ ^]^ that, along with related coherent brightfield (COBRI) microscopy, has managed to approach kHz sampling rates in SPT on cell membranes.^[^ ^17^ ^]^ In ISCAT microscopy, the reflected and backscattered signal originated by the coherent incident light generate an interference figure on the camera sensor.^[^ ^18^ ^]^ The imaging contrast is mainly given by the phase difference term between the incident and reflected field. This results in increased signal‐to‐noise (SNR) ratios, and consequently better localization precision, compared to darkfield and brightfield microscopy.^[^ ^19^ ^]^ Significantly, this increase in SNR has enabled the use of smaller scattering probes,[^17^, ^20^] while also benefitting from the ability to collect very long, nonintermittent, single‐molecule trajectories. Given the relative simplicity of the experimental setup and the data analysis, ISCAT has already been employed to perform SPT experiments on model and cellular membranes.^[^ ^15^, ^17^, ^21^ ^]^

The goals of this study were to 1) fully exploit the ability of ISCAT microscopy to collect long, continuous, single particle trajectories of diffusing lipids at fast 2 kHz acquisition rates, high SNR, and great localization precision in the plasma membrane of live Ptk2 cells, and 2) to develop an unbiased analysis pipeline that is capable of extracting maximum information out of this data. In accordance, we present the results from use of a novel unbiased, statistics driven analysis pipeline applied to the diffusion dynamics of a biotinylated lipid analog tagged with two sizes of highly scattering Au NPs. We used standard 40 nm diameter (Ø40 nm) streptavidin (sAv) coated Au NPs, sAv‐Ø40 nm Au NPs (as have previously been used extensively in high‐speed SPT studies from the Kusumi lab^[^ ^11^ ^]^) and twofold smaller by diameter (i.e., eightfold smaller by volume) sAv‐Ø20 nm Au NPs to label individual biotinylated phospholipid analog, in the plasma membrane of live Ptk2 cells, at physiological conditions, on a custom ISCAT setup with a data acquisition rate of 2 kHz.

The resultant single‐particle trajectories were analyzed by a modified version of our previously published mean squared displacement (MSD) based analysis pipeline for ensemble average trajectory analysis^[^ ^22^ ^]^ that now also enables robust analysis of particle trajectories at the single‐trajectory level. The distinguishing feature of this analysis pipeline is the implementation of a statistics‐driven Bayesian Information Criteria (BIC) method that enables an unbiased determination of the most likely diffusion mode from a set of user‐defined plausible diffusion mode models, as well as the relevant diffusion model parameters for a particular particle trajectory data set. Significantly, this analysis pipeline also directly extracts an estimation of the localization precision from the trajectories of diffusing lipids, the so‐called dynamic localization precision. This is in contrast with the institution of an equivalent static localization precision as pre‐determined from analysis of immobilized lipids or particles, which, as we highlighted here, tended to underestimate the noise and thus overestimate short‐range mobility. An open‐source toolkit to replicate this analysis pipeline has been separately published for the benefit of the community.^[^ ^23^ ^]^ Much related work with developing various machine learning approaches for analyzing single‐molecule trajectories is also underway (see e.g., review in ref. [24]) but there is as of yet no efficient mean for such analysis in the case of highly heterogeneous diffusion data as is expected in the plasma membrane of live cells.

Application of our unbiased analysis pipeline to the experimental data showed that the most likely diffusion model for the ensemble average of tracked single phospholipids in the plasma membrane of live Ptk2 cells, for both gold particle sizes, out of a diffusion model set that consisted of free, confined, anomalous, and transient compartmentalized diffusion, was transient compartmentalized diffusion with an average compartment size of around 100–110 nm. The ensemble average analysis results further suggested that the use of smaller, presumably less invasive sAv‐Ø20 nm Au NPs as compared to larger sAv‐Ø40 nm Au NPs, resulted in increased diffusion rates and weaker confinement, but had no effect on the diffusion modes or on the inferred average compartment size. The employment of the same analysis pipeline at the single‐trajectory level, with the same diffusion models, revealed as expected much more complex behavior with an equal prevalence of transient compartmentalized (40%), and anomalous (40%) diffusion along with examples of both free (7–8%) and confined (13–14%) diffusion, while the resulting fit parameters were broadly distributed. Putting our results in a broader context with previous results, we have adopted a confinement strength (*S*_conf_) metric in combination with simplified Monte Carlo simulations of transient compartmentalized diffusion on a heterogeneous lattice. as previously described.^[^ ^3^ ^]^ We highlight that this allows for straightforward comparison between our experimental results with other related studies from the same or other related techniques. The use of this metric shows that the diffusive motion herein described appears to be neither cell line nor method specific, as demonstrated by comparison with the data from past experiments.^[^ ^2^, ^3^, ^8^, ^22^ ^]^

## 2 Results and Discussion

### 2.1 ISCAT Microscopy Results in Long, Continuous Single Particle Trajectories at an Acquisition Rate of 2 kHz

In this work, we have used a custom‐built ISCAT microscope, previously described in ref. [15], to investigate the lateral diffusion in the apical plasma membrane of live PtK2 cells of artificially incorporated biotinylated phospholipids, DSPE‐PEG2000‐Biotin (1,2‐distearoyl‐sn‐glycero‐3‐phosphoethanolamine with a 2 kDa PEG linker to biotin). We employed two distinct sizes of streptavidin (sAv)‐coated Au NPs: 40 nm in diameter (sAv‐Ø40 nm Au NPs, as had previously been used in related studies in conventional brightfield‐based SPT^[1a]^), and 20 nm in diameter (sAv‐Ø20 nm Au NPs) (Table of Contents Scheme). Using our ISCAT set‐up, we were able to obtain single‐frame image data for both gold NP sizes at sufficient SNR to yield long, continuous, single lipid trajectories at a sampling frequency of 2 kHz (Supplementary Information; **Table** **1**). This validated the ability of ISCAT microscopy to enable the acquisition of high‐speed, long continuous single trajectories of diffusing lipids in the plasma membrane of live cells with twofold smaller by diameter, and thus eightfold smaller by volume, sAv‐Ø20 nm Au NPs that are likely much less invasive than the much larger sAv‐Ø40 nm Au NPs.

**Table 1:** Summary statistics for the detected ISCAT trajectories.

Sample	N_Traj_ a)	Trajectory length (±s.t.d.) (frames)	*N*_Traj_^500^ b)
DSPE‐PEG2000‐biotin/sAv‐Ø20 nm Au NPs on Ptk2 cells	92	1430 ± 970	229
DSPE‐PEG2000‐biotin/sAv‐Ø40 nm Au NPs on Ptk2 cells	165	1510 ± 1040	433
sAv‐Ø20 nm Au NPs immobilized on glass	4	4000 ± 0	32
sAv‐Ø40 nm Au NPs immobilized on glass	4	4000 ± 1	31

a) *N*_Traj_ is the total number of detected ISCAT trajectories.

b) *N*_Traj_^500^ is the total number of equi‐length truncated ISCAT trajectories that were subject to extended quantitative analysis.

**Figure 1:**
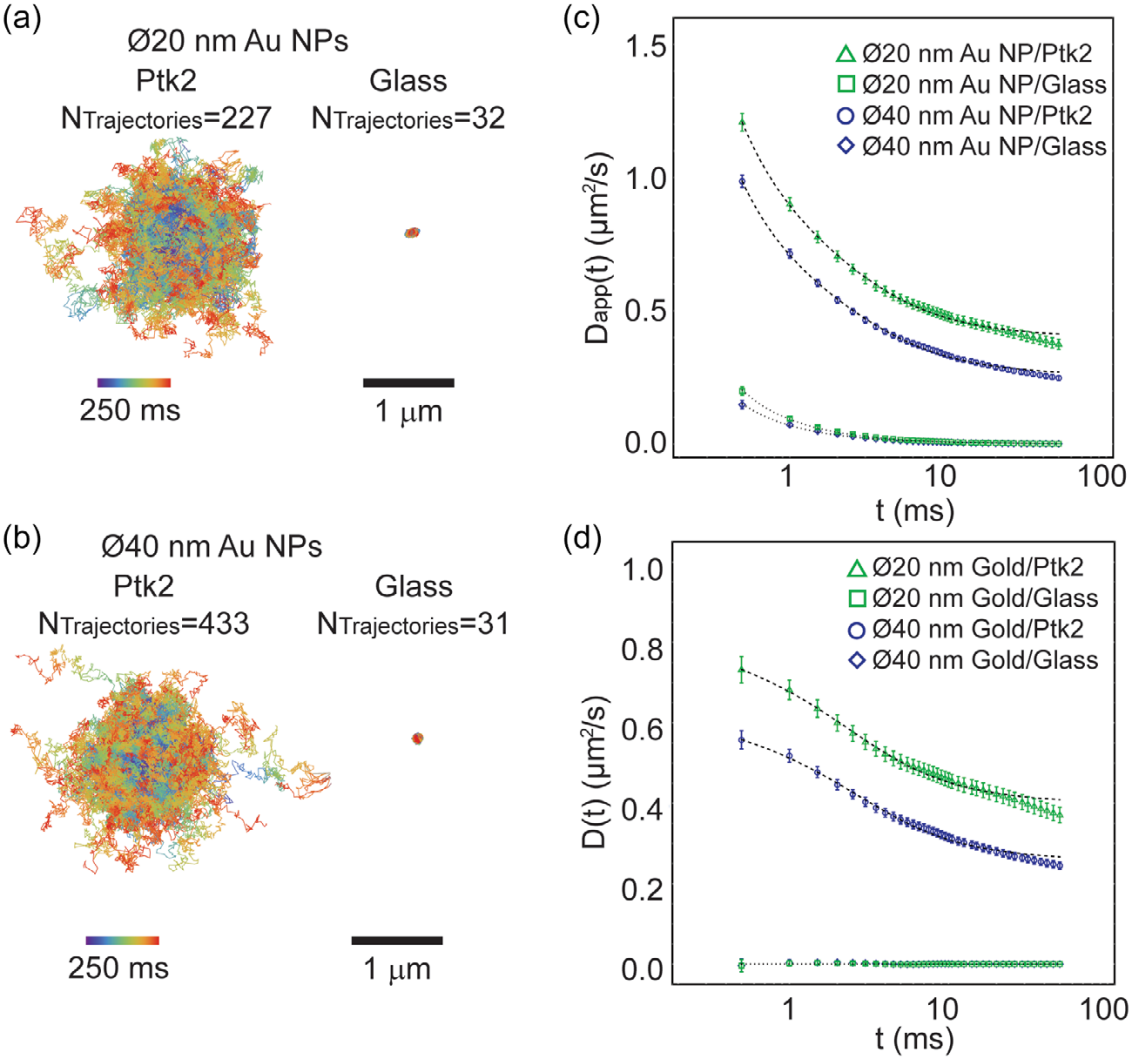
Experimental ISCAT tracking data. a) Superimposed trajectories from time‐lapse image acquisitions of diffusing DSPE‐PEG(2000)‐Biotin lipid analogs in the plasma membrane of Ptk2 cells labelled with sAv‐Ø20 nm Au NPs, and (right) the same Au NPs immobilized on glass (acquisition frame rate = 2 kHz). All trajectories were truncated to 250 ms (*n* = 500 displacements) long segments, remapped to start at the same position. The color scale indicates the time of each localization (violet to red). b) Same as in (a) except that lipid analogs were labeled with sAv‐Ø40 nm Au NPs. c) Comparison between the ensemble average of the *D*_app_(*t*) curves obtained from diffusion of sAv‐Ø20 nm AU NP‐tagged (green triangles) and sAv‐Ø40 nm Au NP‐tagged lipids on (blue circles) in the plasma membrane of live Ptk2 cells, and the same size Au NPs immobilized on glass (Ø20 nm (green squares); Ø40 nm (blue diamonds)). The most likely models of diffusion (dashed lines) all included a dynamic localization uncertainty and were the following for the different samples were: 1) sAv‐Ø20 nm and sAv‐Ø20 nm Au NPs in Ptk2 cells: exact compartmentalized diffusion (Equation 6.2); 2) Ø20 nm Au NPs on glass: immobile (Equation 8); and 3) sAv‐Ø40 nm Au NPs on glass: free diffusion (Equation 3) with a very low diffusion coefficient (dashed lines). The according fit parameters are shown in Table 3. d) The true time‐dependence of the diffusive motion after subtraction of the fitted dynamic localization uncertainty *δ*_*xy*_. Also shown (dashed lines) are the most likely model fits for each data set.

For further downstream analysis, all single trajectories were trimmed to a constant track length of 500 sequential displacements corresponding to a track duration of 250 ms each. This was done to ensure constant statistical sampling of each trajectory type, independent of the diffusion modes, as there was otherwise a trend where trajectories for slow moving or immobile particles were generally much longer than the equivalent trajectories for faster moving particles. The super‐imposed resulting trimmed trajectories are visualized in **Figure** **1**a for sAv‐Ø20 nm Au NPs, and Figure 1b for sAv‐Ø40 nm Au NPs. Related tracks of immobilized Au NPs on a glass coverslip are shown for reference for each probe size. In these plots, all single trajectories were super‐imposed to initiate at the origin while time duration is encoded with a rainbow color scale from time *t* = 0 ms (violet) to *t* = 250 ms (red). The superimposed trajectories show that the gold particle‐labeled DSPE lipids on PtK2 cells diffused away from the center in a broadly symmetrical direction, reflecting the stochastic nature of the long‐term motion of the tracked particles. In contrast, the tracks from stationary gold particles represented the minimal motion which was directly distinguished by our experimental approach. The comparison to stationary gold NPs in this context was ultimately very important as it provided an optimal measure of the microscope system performance in terms of the lower limit of the dynamic localization precision. These datasets were the result of experiments carried over several samples, on two different cell cultures measured months apart from each other.

### 2.2 A Qualitative Analysis of the Ensemble Average ISCAT Trajectory Data Indicates that the Translational Motion is Complex

A qualitative, nonfitting, analysis of the fully processed time‐dependence of the ensemble average translational motion of the ISCAT trajectory data from Figure 1a,b is shown in Figure 1c. In this plot, we followed the same procedure as before^[^ ^22^ ^]^ by calculating the camera‐blur corrected apparent diffusion coefficient, *D*_app_(*t*) for each single‐trajectory from

(1) $$D_{\text{app}}\left(t\right)=\frac{\text{MSD}\left(t\right)}{2\;d\;n\;t_{\text{lag}}\left(1-\frac{2R}{n}\right)}$$

where MSD(*t*) is the calculated conventional time‐averaged mean squared displacement as a function of displacement times *t*, *d* is the dimensionality of the diffusion process, *t*_lag_ is the lag time between successive experimental data points, *n* is an integer that denotes the number successive time steps for a time interval of *t* = *n* *t*_lag_, and *R* is the motion blur correction factor (see extended discussion in Supplementary information). Because the image data in this study were acquired in a single optical plane, we restricted our analysis to diffusion in a 2D plane (i.e., *d* = 2). This approach enabled a direct, nonfitting, visualization of the complexity of the time‐dependence of the translational motion of experimental singe particle trajectory data directly in terms of an apparent diffusion coefficient *D*_app_(*t*).

One such complexity in the case of the data in this paper was a systematic vibration artefact at frequency of 143 Hz (≈8600 rpm) and a constant root mean squared amplitude of less than 10 nm (Supplementary Table 1, Supporting Information). While best experimental procedure is to avoid such artefacts, for the purpose of this work, we showed that this artefact was efficiently eliminated through a simple Fourier filtering procedure as described in the Supplementary information. The data shown in Figure 1c are the resultant vibration‐corrected plots for *D*_app_(*t*), while the same raw data are shown in Supplemental Figure S3a, Supporting Information. In contrast, the data shown in Figure 1a,b have not been corrected for the same vibration artefact as this would further require precise knowledge about the direction and phase of the vibration. Also shown in Supplemental Figure S3, Supporting Information is the ensemble average data in the more conventional format of the time‐dependence of the MSD(*t*), without (Figure S3b, Supporting Information) and with (Figure S3d, Supporting Information) the above vibration correction. It is particular striking that the systematic effect of the vibration was much less visible in the conventional MSD(t) format except in the inserted log‐log format of the control data of immobilized Au NPs.

The qualitative views (Figure 1c) of the ensemble averaged ISCAT trajectory data directly indicated that the observed translational motion of lipids in the plasma membrane of live Ptk2 cells was indeed complex with a gradual slowing of the apparent diffusion coefficient *D*_app_(*t*) at longer times *t*. Furthermore, the average *D*_app_(*t*) of DSPE‐PEG2000‐biotin lipids tagged with the smaller sAv‐Ø20 nm Au NPs in the plasma membrane of PtK2 was greater at all time points than when labelling the same lipids with the larger sAv‐Ø40 nm Au NPs. In addition, the *D*_app_(*t*) format of our ISCAT data also directly illustrates the profound effect of the artificial diffusion‐like component that originated from the finite dynamic localization precision, *δ*_*xy*_. This effect is best shown in Figure 1c for the plots for the immobilized Au NPs at times *t* < 2 ms where this term contributes an artificial diffusion‐like component of *D*_app_(*t*) that converges to zero at time *t* > 2 ms. This effect is also our rationale for referring to the calculated quantity from Equation (1) as an apparent diffusion coefficient, *D*_app_(*t*), as this quantity is affected by experimental measurement noise including effects due to diffusive motion during the camera acquisition time (i.e., camera blur),^[^ ^25^ ^]^ and localization precision.^[^ ^26^ ^]^ As shown in the Supplementary Information, we can express the relationship between the apparent diffusion coefficient *D*_app_(*t*), as calculated from Equation (1), and the true, potentially, time‐dependent diffusion coefficient *D*(*t*) as
(2)

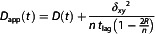

where *δ*_*xy*_ is the localization precision with an assumption that the precision in the *x*‐direction, *δ*_*x*_, and *y*‐direction, *δ*_*y*_, are equal (i.e., *δ*_*x*_ = *δ*_*y*_ = *δ*_*xy*_ (Supplementary information)), and the other terms are the same as before in Equation (1). It is the second term on the right‐hand side of Equation (2) that makes high‐speed tracking experiments very susceptible to misinterpretation of the time‐dependence of the true diffusion coefficient *D*(*t*), as this term diverges in the limit of *t* = *n* *t*_lag_ → 0. The influence of this second term is the reason why even the *D*_app_(*t*) plots in Figure 1c for immobilized Au NPs, as well as the same plots for the Au NPs attached to diffusing lipids, appear to diverge in this time limit. Noteworthy is that this same effect is much less noticeable in the case of the more conventional time‐dependence of MSD(*t*) plots, as shown in Supplementary Figures S3b,d, Supporting Information, since the same term in this case is given by a constant offset with a typical minimal magnitude of 4 *δ*_*xy*_^2^/(1 − 2*R*/*n*). Yet, as already shown in Figure 1c, the effect of this term in terms of the time‐dependence of the apparent diffusion coefficient *D*_app_(*t*) is potentially profound.

### 2.3 Establishment of an Unbiased, Statistics‐Driven Quantitative Workflow for Robust Analysis of ISCAT Single Particle Trajectories

We next turned to establish the foundation for a robust, nonbiased, statistics‐driven quantitative analysis pipeline that is also applicable at the single‐molecule trajectory level. This work is an extension to our previously published analysis pipeline for the analysis of ensemble average SPT data.^[^ ^22^ ^]^ Whereas a typical justification for the applications of single‐molecule tracking techniques is the ability to explore heterogeneity among single‐molecule behaviors, we note that this is in practice very rarely fully exploited. A great advantage of our ISCAT trajectory data is that such an analysis can readily be applied, since ISCAT enables the collection of long, continuous single particle trajectories.

The important feature of our analysis method is that we performed an unbiased analysis of the experimental ISCAT trajectory data, as calculated by Equation (1), by weighted, nonlinear fitting of the data using Equation (2) to a set of plausible diffusion mode models after which we used the BIC to determine the most likely model out of the evaluated set of plausible models (Supporting Information). In simplistic terms, this data analysis approach enabled an unbiased classification of the diffusion mode of an experimental trajectory data set by asking the question: what is the simplest diffusion mode fit model, from a set of plausible user‐defined models, that best describes the experimental data set? In answering this question, the BIC approach of this analysis pipeline applied a rewarding term that was dependent on minimization of the residual sums of square between the experimental data set and the best fit. Yet, hereby more complex data models were penalized from a term that scaled linearly with the number of free parameters in the fit model (Supplementary Information).

In our analysis workflow, we substituted a range of expressions of *D*(*t*) corresponding to different plausible diffusion mode models (**Table** **2**),^[^ ^27^ ^]^ and subsequently performed a nonlinear weighted fit to the ensemble‐averaged *D*_app_(*t*) data (Figure 1c). In these fits, the localization precision *δ*_*xy*_, was included as a free parameter in the fitting routine. By this approach, we were able to directly extract an estimate of the localization precision from the trajectory data of both moving particles, the so‐called “dynamic” localization precision, as was the case for tagged lipids, and for immobile particles, the so‐called “static” localization precision, as was the case for immobilized NPs on a glass substrate. This distinction was especially important for high‐speed tracking experiments because 1) the “dynamic” localization precision is expected to always be greater than the “static” localization precision, and 2) there is a divergence of the diffusive‐like term that is dependent on the localization precision as shown in Equation (2) and the associated discussion. Furthermore, the time‐dependence of the apparent diffusion coefficient *D*_app_(*t*) data in Figure 1c is suggestive of a change of state from faster diffusion to slower diffusion at time scales of *t* > 10 ms. To ensure that our analysis approach was able to accurately capture this transition, we sampled available data points on a log‐time scale such that the many points at longer time lags were not overwhelmingly weighted compared to the much fewer points at the earliest time lags (Supplementary Information).

**Table 2:** Plausible diffusion mode models.

Diffusion mode model	Diffusion coefficient $D\left(t\right)=\frac{\text{MSD}\left(t\right)}{4t}$	Eq.
Free (Brownian)	$D\left(t\right)=D_{M}$	3
Directed motion	$D\left(t\right)=\frac{v^{2}\cdot t}{4}$	4
Confined (approximate)	$D\left(t\right)=\frac{L^{2}}{12t}\left[1-\exp\left(-\frac{12D_{\mu }t}{L^{2}}\right)\right]$	5.1
Confined (exact)^[^ ^29^ ^]^	$D\left(t\right)=\frac{L^{2}}{12t}\left(1-\frac{96}{\pi^{4}}\sum_{k=1,3,5,\ldots }^{\infty }\frac{1}{k^{4}}\exp\left(-{\left(\frac{k\pi }{L}\right)}^{2}D_{\mu }t\right)\right)$	5.2
Transient compartmentalized (approximate)^[^ ^7^, ^28^ ^]^	$D\left(t\right)=D_{M}+\frac{D_{\mu }-D_{M}}{D_{\mu }}\frac{L^{2}}{12t}\left[1-\exp\left(-\frac{12D_{\mu }t}{L^{2}}\right)\right]$	6.1
Transient compartmentalized (exact)^[^ ^29^ ^]^	$D\left(t\right)=D_{M}+\frac{D_{\mu }-D_{M}}{D_{\mu }}\frac{L^{2}}{12t}\left(1-\frac{96}{\pi^{4}}\sum_{k=1,3,5,..}^{\infty }\frac{1}{k^{4}}\exp\left(-{\left(\frac{k\pi }{L}\right)}^{2}D_{\mu }t\right)\right)$	6.2
Anomalous	$D\left(t\right)=\Gamma t^{\alpha -1}$	7
Immobile	D(t)=0	8

The diffusion mode models that were used in this work are shown in Table 2. These include models for normal (Brownian) diffusion (Equation 3), directed motion (Equation 4), two models each for confined (Equation 5.1 and 5.2) and transient compartmentalized (hop) diffusion (Equation 6.1 and 6.2), the approximate versions (Equation 5.1 and 6.1),^[^ ^28^ ^]^ and the exact expression for confined diffusion within permeable square corrals of size *L* (Equation 5.2 and 6.2).^[^ ^7^, ^29^ ^]^ Notably, Equation (5.2) and (6.2) contain infinite sums, which cannot be fit to data analytically. Thus, for this study, we have restricted these equations to the case in which *k* ≤ 39, which converges well to the approximate forms (Equation 5.1 and 6.1) for *t* > 0.5 ms, although more terms would be required for fitting to ISCAT data acquired at a faster sampling acquisition. We have also included a model for anomalous diffusion (Equation 7). The interpretation of anomalous diffusion in terms of the inferred nano‐organization of the plasma membrane is much more challenging than for the other diffusion mode models in Table 2. Unlike transient compartmentalized diffusion, which points directly at a precise mechanism, anomalous subdiffusion in the plasma membrane can originate from, for example, diffusion in a heterogeneous environment due to a mix of obstacles, binding, and crowding,^[^ ^30^ ^]^ or alternatively an infinite arrangement of transient compartments but with a very broad size distribution, and an equally very broad distribution of transient confinement times.^[^ ^28^, ^30^, ^31^ ^]^ Alternatively, anomalous subdiffusion could highlight lipid trajectories that underwent a change in the diffusion mode from free to confined, or similar. Finally, we have also included a diffusion mode model for immobile particles (Equation 8). The diffusion mode models in Table 2 have been scaled such the parameter *D*_M_ represents the long‐term, time‐independent, possibly intercompartment diffusion coefficient, while *D*_μ_ represents the unhindered intracompartment diffusion coefficient of the molecule inside a specific compartment.

To increase robustness of our weighted, nonlinear fitting routines, we further required that all free parameters in a given model fit converged to nonzero value with a significance level of *p* < 0.05. Using these two metrics, the most likely model to describe each trajectory, therefore, must satisfy two main conditions: 1) minimizing the BIC (i.e., the highest Relative Likelihood), and 2) that all fit parameters were significantly different than zero. This analysis approach provided an unbiased, statistics‐driven method to determine the most likely diffusion mode from a pool of user‐defined models, and of course the corresponding fit parameters

### 2.4 A Quantitative Analysis of the ISCAT Trajectory Data through our Unbiased, Statistics‐Driven Analysis Pipeline Indicates that the Most Likely Ensemble Average Diffusion Mode is Transient Compartmentalized Diffusion

We first applied our improved analysis workflow to the ensemble average ISCAT trajectory *D*_app_(*t*) data, as shown in Figure 1c. One of the challenges in this analysis is that there is no definite consensus regarding the amount of points of a MSD curve to be analyzed to describe the diffusion motion of the tracked particle, only a “rule of thumb” prescribing to use less than 25% of the total data points.^[^ ^32^ ^]^ For this reason, we evaluated the dependence on the fit results upon the analysis time range for six different time intervals, 0.5 ≤ *t* ≤ *T* ms, where *T* was set to 5, 10, 25, 50, 75, and 100 ms, respectively. At the employed sampling frequency of 2 kHz, this corresponds to 10 (2%), 20 (4%), 50 (10%), 100 (20%), 150 (30%), and 200 (40%) points of the analyzed truncated 500 localization long trajectory segments. From this analysis, we found that the most likely model to describe the time‐dependence of the ensemble averages *D*_app_(*t*) curves, at all six different time intervals, for both the sAv‐Ø20 nm and sAv‐Ø40 nm Au NP‐tagged lipids was the exact compartmentalized diffusion model (Equation 6.2). The fits to the approximate version of the same model (Equation 6.1) also resulted in high relative likelihood values (*R* > 0.7), suggesting that this model also accurately described these data sets, albeit slightly worse than the exact solutions. This result, that the mode of diffusion was not affected by the probe size is consistent with our previous findings in related studies in supported lipid bilayers for ISCAT measurements acquired at 100 Hz.^[^ ^15^ ^]^

**Figure 2:**
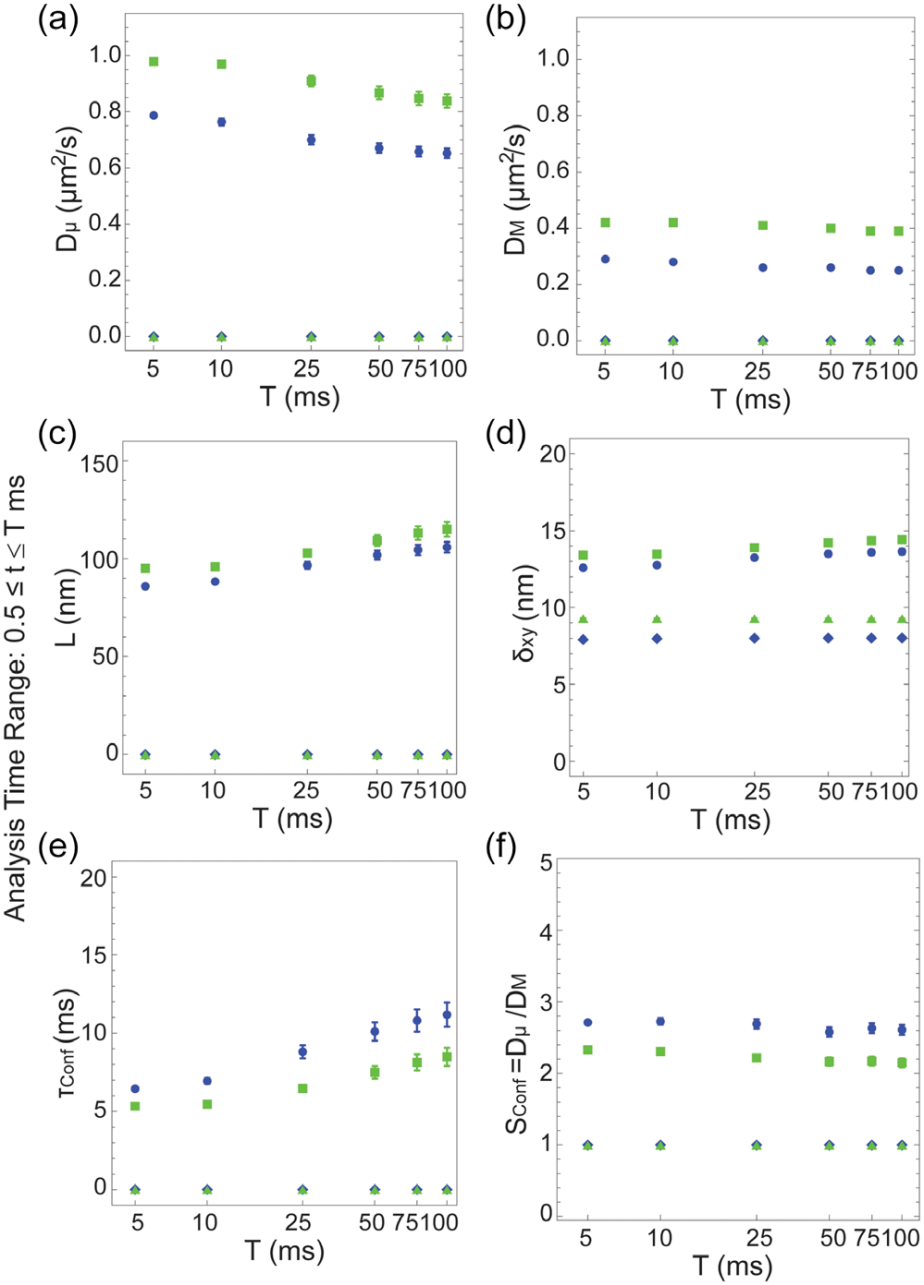
Comparison of fit parameters from the analysis of the ensemble average *D*_app_(*t*) curves. The ensemble average *D*_app_(*t*) curves obtained from the trajectories of sAv‐Ø20 nm Au NP‐tagged DSPE lipids (green rectangles), and sAv‐Ø40 nm Au NP‐tagged DSPE lipids (blue circles) in the plasma membrane of live Ptk2 cells, and the same NPs immobilized on glass (sAv‐Ø20 nm Au NPs (green triangles); sAv‐Ø40 nm Au NPs (blue diamonds)) were analyzed at different time intervals (0.5 ≤ *t* ≤ *T* with *T* = 5, 10, 25, 50, 75, and 100 ms). Plotted are the various fit parameters, obtained from the most likely diffusion model to the data, and the resulting confinement strength metrics. The values shown are: a) the intracompartmental diffusion coefficient, *D*_μ_, b) the intercompartmental diffusion coefficient *D*_M_, c) the confinement size *L*, d) the dynamic localization precision *δ*_*xy*_, e) the confinement time *τ*_Conf_ = *L* ^2^/(4 *D*_M_), and f) the confinement strength *S*_Conf_ = *D*_μ_/*D*_M_.

We also analyzed the control data for immobilized Au NPs using the same approach. This analysis showed that the most likely diffusion mode for immobilized sAv‐Ø20 nm Au NPs, at all six time intervals, was the model for immobile particles (Equation 8). The data for immobilized sAv‐Ø40 nm Au NPs, at all six time intervals, were instead best described by the model for free (Brownian) diffusion (Equation 3), but with a very low diffusion coefficient (*D* < 0.003 μm^2^ s^−1^; Supplemental Table S5, Supporting Information). A full summary of the fit parameters, for every time range, is given in Supplemental Table S3–S6, Supporting Information. The fit parameters from the most likely diffusion modes, at all six time intervals, are plotted in **Figure** **2**.

Figure 2 includes two additional metrics that can be calculated from the fit parameters which are useful for characterizing transient compartmentalization. These are the confinement time, *τ*_Conf_, that a molecule spends on average within a squared compartment with an area of on average *L* ^2^ before escaping to an adjacent compartment^[1a]^ (Figure 2e)

(9) $$\tau_{\text{Conf}}=\frac{L^{2}}{4\cdot D_{M}}$$

and the confinement strength, *S*_Conf_, which is simply the ratio of the faster intracompartmental diffusion coefficient *D*_μ_ and the slower intercompartmental diffusion coefficient *D*_M_^[^ ^7^ ^]^ (Figure 2f)

(10) $$S_{\text{Conf}}=\frac{D_{\mu }}{D_{M}}$$

Useful extreme limits of these metrics are for free Brownian diffusion, *τ*_Conf_ = 0 as *L* = 0, and *S*_Conf_ = 1 as *D*_μ_ = *D*_M_, and for confined diffusion, *τ*_Conf_ = ∞ and *S*_Conf_ = ∞ as *D*_M_ = 0. In the case of transient compartmentalized diffusion, the magnitudes of these metrics relative to the above limits are useful for understanding the strength of the confinement. Neither of these two metrics is defined in the case of anomalous diffusion, although one could consider an alternate form of Equation (10) to calculate an equivalent confinement strength from at two specific time‐points, *t*_1_ and *t*_2_, such as, e.g., at *t*_1_ = 1 ms and *t*_2_ = 50 ms from *S*_Conf_ = (*t*_1_/*t*_2_)^*α*−1^. This revised definition of the confinement strength in the case of anomalous diffusion in the limit as *t*_2_ → ∞ is the same as in the case of confined diffusion of *S*_Conf_ → ∞.

The magnitudes of the various fit parameters in Figure 2 (and Supplementary Table S3–S6, Supporting Information) from the most likely fits to the ensemble average data are largely invariant, across the six analysis time intervals, with only a modest variation on the order of 10% for the intracompartmental diffusion coefficient, *D*_μ_, the intercompartmental diffusion coefficient *D*_M_, and the compartment size, *L* (Supplementary Table S3 and S4, Supporting Information). Thus, for simplicity for the subsequent discussion, we have used the fit results for the intermediate time interval of 0.5 ≤ *t* ≤ 50 ms (**Table** **3**) for the rest of the discussion. This intermediate analysis time interval corresponds to ≈20% of the total data points in each trajectory segment considered by our analysis, putting this choice below the aforementioned “rule of thumb”.^[^ ^32^ ^]^ The actual fit results for most likely diffusion mode model for each condition are included, as dashed lines in Figure 1c. For completeness, the fit parameters for all time intervals are shown in the Supplementary Information.

**Table 3:** Ensemble average analysis fit parameters for an analysis time interval 0.5 ≤ *t* ≤ 50 ms.

Sample	*D*_μ_ [μm^2^ s^−1^]	*D*_M_ [μm^2^ s^−1^]	*L* [nm]	*δ*_*xy*_ [nm]	*τ*_Conf_ [ms]	*S*_Conf_
sAv‐Ø20 nm Au NPs Ptk2	0.87 ± 0.02a)	0.4 ± 0.00	110 ± 3.0	14.2 ± 0.2	7.5 ± 0.4b)	2.2 ± 0.0
sAv‐Ø20 nm Au NPs glass	–	–	–	9.3 ± 0.1	–	1.0
sAv‐Ø40 nm Au NPs Ptk2	0.67 ± 0.02	0.26 ± 0.00	100 ± 2.3	13.5 ± 0.2	10 ± 0.6	2.6 ± 0.1
sAv‐Ø40 nm Au NPs glass	–	0.001 ± 0.0005	–	8.0 ± 0.1	–	1.0

a) The errors reported for *D*_μ_, *D*_M_, *L*, and *δ*_*xy*_ refer to the standard error of the fit.

b) The error for *τ*_Conf_ and *S*_Conf_ are the standard deviations as obtained with standard rules of error propagation.

The fit parameter results confirmed that high‐speed ISCAT tracking data of lipids in the plasma membrane of live Ptk2 cells can be performed at very high localization precision of *δ*_*xy*_ < 15 nm, even in the case of the smaller Ø20 nm Au NPs. These smaller Au NPs are 2× smaller in diameter (and thus 8× smaller in volume) than the larger Ø40 nm Au NPs that are typically used for conventional scattering‐based SPT tracking experiments.^[1a]^ With our ISCAT setup, we indeed observed only a slight loss of localization precision (≈5% for live cell data, and <15% for immobilized NPs) for data acquired with smaller Ø20 nm as compared to larger Ø40 nm Au NPs. The fit parameter results further confirmed, as expected, that the dynamic localization error, as determined from analysis of tagged lipids, is greater than the static localization error, as determined from the analysis of the immobilized Au NPs for both Au NP sizes (Ø20 and Ø40 nm).

From the magnitudes of the localization precision *δ*_*xy*_ for each data set, it is possible to extract the real time‐dependence of the diffusion data, *D*(*t*) by subtraction of the localization error term in Equation (2). These plots are shown in Figure 1d. Satisfyingly, these corrected plots show, as expected, that the control data for the immobilized particles are indeed immobile (i.e., *D* ≪ 0.01 μm^2^ s^−1^) for the entire time interval, while the appearance of the localization precision corrected experimental *D*_app_(*t*) data for the diffusing lipids has in the limit of decreasing time intervals, *t*, been transformed from diverging curves (Figure 1c) to curves that hint at convergence in the same limit (Figure 1d). The fit results for the localization precision also established the lower limit for which our ISCAT system was able to detect transient compartmentalization in the plasma membrane. As shown in Table 3 (and more extensively in Table S2, Supporting Information), these limits were *δ*_*xy*_ = <10 nm for stationary lipids, as inferred from single particle tracks of immobile Au NPs, and *δ*_*xy*_ < 15 nm for transiently mobile lipids, as inferred from trajectories of tagged lipids in the plasma membrane of Ptk2 cells. In terms of areas of nanocompartments in the plasma membrane of live cells, this would correspond to a lower detection limit of the compartment size of *L* = *δ*_*xy*_ ≈ 15 nm, and *L* ^2^ > ≈225 nm^2^ for transiently mobile lipids.

The localization precision‐corrected plots, as shown in Figure 1d, and the fit parameters *D*_μ_, *D*_M_, and *L* in Table 3 clearly confirmed that our results were dependent on the size of the Au NP. As such, the ensemble average diffusion coefficient of the lipids that were tagged with the smaller Ø20 nm Au NPs was slightly faster, at all times, both within compartments highlighted by the parameter *D*_μ_, and between compartments, highlighted by *D*_M_, than lipids that were tagged with the larger Ø40 nm Au NPs. In contrast, the ensemble average compartment size *L* was almost the same (*L* = 110 nm for smaller Ø20 nm AU NPs versus *L* = 100 nm for the larger Ø40 nm AU NPs). Notably, these ensemble average compartments sizes were about tenfold larger than our lower detection limits as discussed above.

The two additional metrics in Figure 2, the average confinement time *τ*_Conf_ and the confinement strength *S*_Conf_, both further suggest that lipid diffusion in the plasma membrane was indeed affected by the probe characteristic where both the average confinement time *τ*_Conf_ and the confinement strength *S*_Conf_ were slightly lower for the smaller Ø20 nm Au NPs than for the larger Ø40 nm Au NPs (*τ*_Conf_ = 7.5 ms versus 10 ms, and *S*_Conf_ = 2.2 versus 2.6), The ensemble average values for the transient confinement times, *τ*_Conf_, thus suggest that the diffusing lipids encountered the confining compartment barriers several times before hopping across to an adjacent compartment. From a simple back of the envelope calculation in support of this, we note that the mean step length for ensemble average intra‐compartmental diffusion within the confining compartment barriers in two dimensions was (4 *D*_μ_ *t*)^0.5^, and thus with *D*_μ_ ≈ 0.7–0.9 μm^2^ s^−1^ (Table 3) *L*/2 ≈ 50 nm for *t* = 1 ms. This would mean that a lipid Au NP complex that originated at the center of a confining compartment of size *L* = 100 nm, and that diffused exactly back and forth from the center to the confining compartment to the compartment barrier for time duration equal to the average confinement time of *τ*_Conf_ = 7.5–10 ms would encounter the confining barrier for a minimum of 4–5 times before escaping (or hopping) to an adjacent compartment.

Collectively, these results suggest that the inferred nano‐organization of the live plasma membrane was not affected by the Au NP size, as both the most likely diffusion mode as well as the compartment size were unaltered. However, lipids that were tagged with the larger Ø40 nm Au NPs diffused slightly slower (*D*_μ_ and *D*_M_), were confined slightly longer (*τ*_Conf_) within the compartments, and thus were slightly more confined (*S*_Conf_) than lipids that were tagged with the smaller Ø20 nm Au NPs. The transient confinement that we observed with both sizes of Au NPs was much more subtle than initial high‐speed SPT measurements acquired at much faster frame rates, which relied on subtracting the static localization precision from measurements on immobilized Au NPs.^[1a]^ This more subtle transient confinement is fully consistent with relatively recent measurements using high‐speed SPT and STED‐FCS.[^1^, ^22^]

### 2.5 Application of Unbiased, Statistics‐Driven Quantitative Workflow for Robust Analysis of Single Particle Trajectories Suggests that the Nano‐Organization of the Plasma Membrane is Very Heterogeneous

The presented analysis workflow to the ensemble average ISCAT trajectory *D*_app_(*t*) data should also provide an efficient way to evaluate the most likely diffusion model at the single‐trajectory level. This would enable a much more in‐depth look at the heterogeneity of the lipid diffusion, and hence, the inferred nano‐organization in the plasma membrane of live Ptk2 cells. Importantly, *D*_app_(*t*) curves obtained from a single trajectory are in general affected by much larger stochastic noise, as compared to ensemble average trajectory analysis, since the number of displacements at each time interval, *t*, are fewer. In this work, we addressed this issue first by minimizing the number of free parameters of the fits to Equation (2) by fixing the dynamic localization precision *δ*_*xy*_ to the value obtained from the corresponding ensemble average analysis. Second, we required that a model fit must have a coefficient of variation *R* ^2 ^> 0.9 to be included in the final analysis. Finally, we used a reduced pool of diffusion models from Table 2 to fit to the single trajectories to simplify the comparison, namely, free (Brownian) diffusion (Equation 3), directed motion (Equation 4), exact confined diffusion (Equation 5.2), exact transient compartmentalized diffusion (Equation 6.2), anomalous diffusion (Equation 7), and immobile particles (Equation 8).

**Figure 3:**
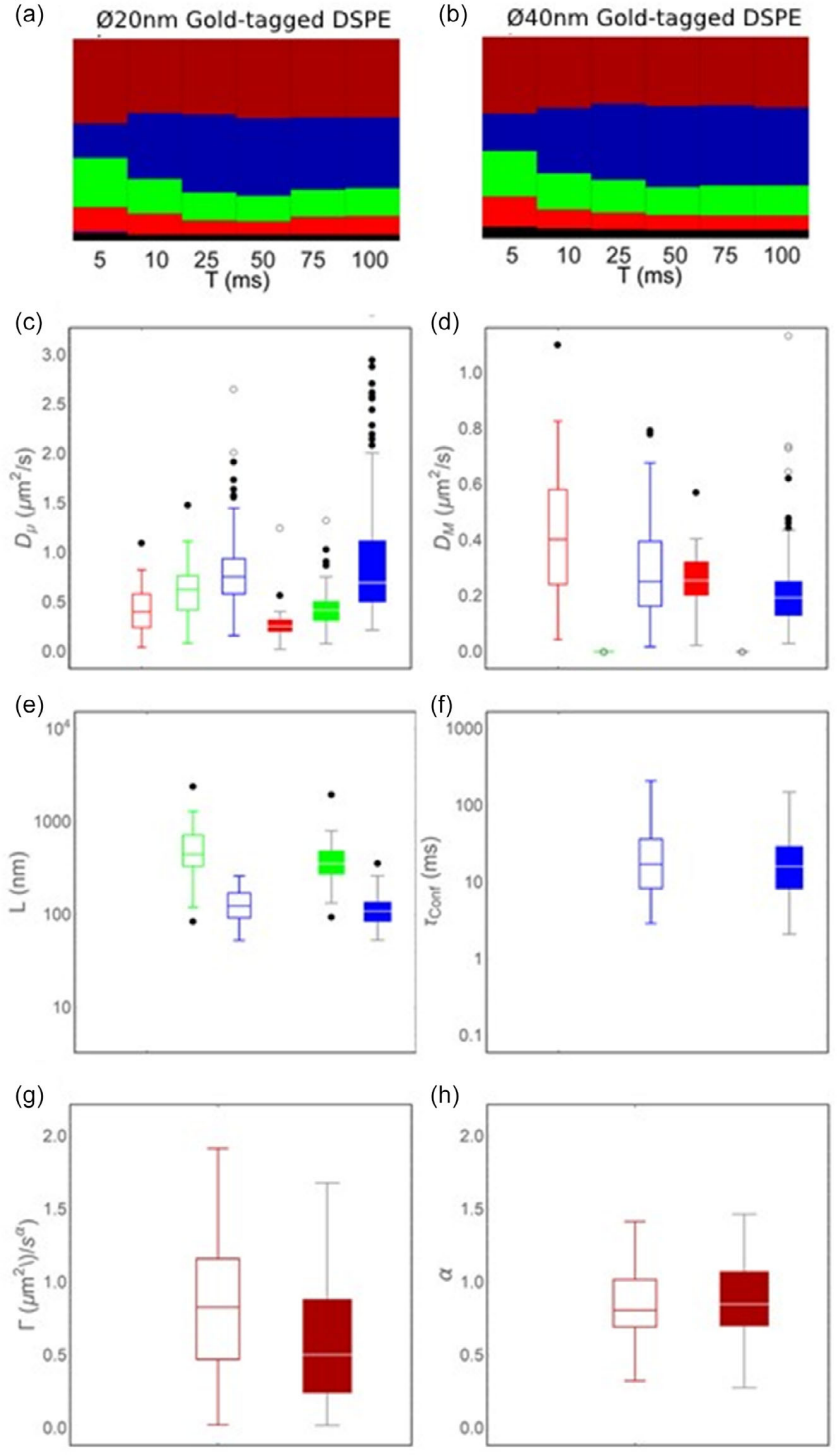
Fit results for the single‐trajectory analysis. a,b) Visual representation of the relative fractions of the most likely diffusion model at different analysis time ranges 0.5 ≤ *t* ≤ *T*), for lipids labelled with (a) Ø20 nm Au NPs, and (b) Ø40 nm Au NPs. The fit models are: anomalous (Equation 7, dark red), exact transient compartmentalized (Equation 6.2, blue), exact confined (Equation 5.2, green), and free (Equation 3, bright red) diffusion. Also shown (black) are the fraction of trajectories for which no model offered a satisfactory solution with the required fit quality metrics. c–h) Fit parameter values from the single‐trajectory fits for the most likely diffusion mode models for each single trajectory. The data are displayed in box and whiskers format. The empty bars are for the fit parameters for lipids labeled with Ø20 nm Au NPs, and the solid bars are for data with Ø40 nm Au NPs. The models and according colors are the same as in (a) and (b). c,d) Intracompartmental diffusion coefficient *D*_μ_ (c) intercompartmental diffusion coefficient *D*_M_ (d) for free (bright red, shown are the same values of *D*_M_ in both panels for comparison), exact confined (green, values of *D*_M_ = 0 μm^2^ s^−1^ as by definition are still plotted in panel (d), and exact transient compartmentalized (blue) diffusion. e) Compartment size *L*, and f) confinement time *τ*_Conf_. as only given for exact confined (green) and exact transient compartmentalized (blue) diffusion. g) Transport coefficient Γ and h) anomaly coefficient *α* fit parameters as only given for anomalous diffusion (dark red). The fit parameters are also summarized in Table 4.

Similarly to the ensemble average analysis, we first evaluated the dependence of the fit results upon the analysis time range for six different time intervals, 0.5 ≤ *t* ≤ *T* ms, where *T* was set to respectively 5, 10, 25, 50, 75, and 100 ms. This analysis showed that the relative fractions of the most likely diffusion mode models at the single‐trajectory level for the lateral motion of biotinylated phospholipids DSPE‐PEG2000‐Biotin in the plasma membrane of live Ptk2 cells, for both sizes of Au NPs, were roughly constant in the analysis time window of 0.5 ≤ *t* ≤ *T* ms for 25 ≤ *T* ≤ 100 ms (**Figure** **3**a,b). In contrast, the confined diffusion mode was much more prevalent at the expense of transient compartmentalized diffusion at the shorter analysis time windows of 0.5 ≤ *t* ≤ *T* ms for 5 ≤ *T* ≤ 10 ms. This suggests that these shorter analysis time windows were not sufficient to fully capture the transition from faster intra‐compartmental diffusion to slower inter‐compartmental diffusion for analysis at the single‐trajectory level. For this reason, we chose, for the purpose of the subsequent discussion, to focus on the fit results for the intermediate time interval of 0.5 ≤ *t* ≤ 50 ms (Figure 3c–h, and **Table** **4**). At this time window, we found that our unbiased analysis approach yielded successful fits for 222 out of 229 (97%) single trajectories of lipids labelled with the smaller Ø20 nm Au NPs, and 415 out of 433 trajectories (96%) for lipids labelled with the larger Ø40 nm Au NPs. This suggests that our unbiased analysis approach was indeed also successfully applicable for analysis at the single‐trajectory level.

**Table 4:** Single‐trajectory analysis fit parameter values (analysis time range 0.5 ≤ *t* ≤ 50 ms).

Au NP [nm]	Diffusion mode	*N* [%]a)	Fit parameters [mean ± s.t.d.]			Metrics [mean ± s.t.d.]	
			*D* [μm^2^ s^−1^]**			*S*_conf_	*τ*_Conf_ [ms]
Ø20	Free (Equation 3)	15 (6.8%)	0.43 (±0.30)			1.0	0
Ø40		32 (7.7%)	0.29 (±0.20)			1.0	0
			*D*_μ_ [μm^2^ s^−1^]**	*D*_M_ [μm^2^ s^−1^]	*L* [nm]**	*S*_conf_	*τ*_Conf_ [ms]
Ø20	Confined (Equation 5.2)	29 (13.1%)	0.61 (±0.31)	0	580 (±450)	∞	∞
Ø40		60 (14.4%)	0.44 (±0.22)	0	400 (±250)	∞	∞
			*D*_μ_ [μm^2^ s^−1^]	*D*_M_ [μm^2^ s^−1^]**	*L* [nm]**	*S*_conf_ **	*τ*_Conf_ [ms]
Ø20	Transient compartmentalized (Equation 6.2)	88 (39.6%)	0.84 (±0.42)	0.29 (±0.18)	130 (±50)	2.7 (±2.2)	28 (±33)
Ø40		174 (39.6%)	0.90 (±0.60)	0.21 (±0.14)	120 (±40)	4.5 (±3.8)	24 (±25)
			Γ [μm^2^ s^−α^]**	*α*		Alt. *S*_conf_ b), c)	*τ*_Conf_ [ms]
Ø20	Anomalous (Equation 7)	90 (40.5%)	0.82 (±0.43)	0.83 (±0.21)		2.7 (±2.2)	n.d.^3^
Ø40		149 (35.9%)	0.57 (±0.40)	0.87 (±0.25)		3.1 (±3.0)	n.d.^3^

a) Number of single particle trajectories, and the fraction of the total number of 500‐localization segments that it represents.

b) Alternative definition of confinement strength for anomalous diffusion as *S*_Conf_ = (*t*_1_/*t*_2_)^*α*−1^ with *t*_1_ = 1 ms and *t*_2_ = 50 ms.

c) n.d. (not defined).

** Fit parameters whose distribution is different according to the Kolmogorov–Smirnov test, with a level of significance *p* < 0.05.

The single‐trajectory analysis results showcased the heterogeneous nature of lipid diffusion, and by inference the heterogeneous nano‐organization in the plasma membrane of live Ptk2 cells. This was apparent from the distribution of the most likely diffusion modes at the level of single trajectories where we observed an equal prevalence of transient compartmentalized (Equation 6.2), and anomalous (Equation 7) (40% each) diffusion along with a subset of free Brownian, (Equation 3; 7–8%) and confined (Equation 5.2; 13–14%) diffusion (Figure 3a,b). Interestingly, consistent both with the above ensemble average analysis, and our previous work,^[^ ^15^ ^]^ the distributions of most likely diffusion modes were again largely unaffected by the size of the Au NPs also at the single‐trajectory level. Notably the magnitudes of the various fit parameters, for both sizes of Au NPs, were characterized by very broad distributions of values (Figure 3c–h, and Table 4), and the magnitudes of the fit parameters within each of these subsets of diffusion modes were, just as in the case of the ensemble average analysis, affected by the Au NP size.

To fully explore the heterogeneity amongst our ISCAT single‐trajectory lipid diffusion datasets, we performed the Kolmogorov–Smirnov (KS) statistical test across datasets, i.e., between the distributions of the values of the fit parameters for the same diffusion mode models but different sizes of Au NPs. The result of this statistical comparison was much less clear‐cut than for the ensemble average analysis. Still, we here summarize the different tendencies of the fit parameters of the different diffusion models as extracted from the single‐trajectory analysis.

In terms of distinct compartment sizes *L*, included in the transient compartmentalized and confined diffusion models, our single‐trajectory analysis results suggested that there are two distinct layers of compartmentalization in the plasma membrane of live Ptk2 cells (Figure 3e, Table 4). This is in full agreement with earlier SPT studies employing large Ø40 nm Au NPs.^[1a]^ One level of compartmentalization was highlighted by the 13–14% fraction of trajectories that were classified as confined diffusion. For this subset of trajectories, the compartment size for lipids labelled with the smaller Ø20 nm Au NPs was <*L*> = 580 ± 450 nm (where <fit parameter> henceforth represents the average and the error term (±) is the standard deviation), and for lipids labelled with the larger Ø40 nm Au NPs was <*L*> = 400 ± 250 nm. The second level of compartmentalization was highlighted by the ≈40% fraction of trajectories that were best described as transient compartmentalized diffusion. The compartment size in this case was <*L*> = 130 ± 50 nm for lipids labelled with the smaller Ø20 nm Au NPs, and <*L*> = 120 ± 40 nm for lipids labelled with the larger Ø40 nm Au NPs. The latter values of around 120–130 nm closely matched the results of compartment sizes of the respective ensemble average analysis. However, the distributions of values of compartment sizes from the single‐trajectory analyses were significantly different (*p* < 0.05) for lipids labelled with the two sizes of Au NPs (and that for both subsets of modes of diffusion, Table 4). This suggests that the Au NPs affected the inferred nano‐organization of the plasma membrane, although at least in the case of the smaller compartment size the change in average compartment size for the two sizes of Au NP was minimal (<*L*> = 130 ± 50 nm for Ø20 nm Au NPs vs <*L*> = 120 ± 40 nm for Ø40 nm Au NPs, compared to 580 ± 450 nm and <*L*> = 400 ± 250 nm). The presence of trajectories classified as anomalous diffusion (40%) could also indicate additional transient nanocompartmentalization but with a broad range of compartment sizes and transient confinement times, but other interpretations such as lipids that underwent a change in the diffusion mode during the duration of the truncated 100 ms trajectories could also be possible.

In terms of the rate of diffusion extracted from the single‐trajectory analysis, we found, largely consistent with ensemble average analysis, that the lipids that had been labelled with the smaller Ø20 nm Au NPs as compared to the larger Ø40 nm Au NPs were generally significantly (*p* < 0.05) faster. In this case, significantly (*p* < 0.05) faster diffusion was observed for the subset of trajectories best described by the following diffusion modes (Figure 3c,d,g,h, Table 4): 1) diffusion coefficient *D*_M_ within free diffusion; 2) intra‐compartmental diffusion coefficient *D*_μ_ within confined diffusion, 3) inter‐compartmental diffusion coefficient *D*_M_ within transient compartmentalized diffusion, and 4) transport coefficient Γ within anomalous diffusion, although in this case there was no significant difference between the anomaly coefficient *α*. The lone exception (*p* > 0.05) to this general observation was the intra‐compartmental diffusion coefficient *D*_μ_ for the subset of trajectories best described as transient compartmentalized diffusion, where lipids were similarly fast for both AU NP label.

When generally comparing the diffusion coefficients of the different diffusion modes for both label sizes, we noted the following order (Figure 3c, Table 4): the intracompartmental diffusion coefficient *D*_μ_^Transient Compartmentalization^ of trajectories best described as transient compartmentalized diffusion was faster than the intra‐compartmental diffusion coefficient *D*_μ_^Confined^ for the subset of trajectories best described as confined diffusion, which was again faster than the free diffusion coefficient *D*_M_^Free^ for the subset of trajectories best described by free diffusion, i.e., *D*_μ_^Transient Compartmentalization^ > *D*_μ_^Confined^ > *D*_M_^Free^. We interpreted this as the fact, that only if compartmentalized diffusion could be resolved on a single‐trajectory level, we could highlight “hopping” between the compartments of around 120–130 nm in diameter with a clearly resolvable diffusion coefficient within the compartments in the range of 0.9 μm^2^ s^−1^, which was much less than the values of 5–10 μm^2^ s^−1^ from previous Au NP labeled and scattering‐based single particle tracking analysis^[1a]^ (following our aforementioned accurate dynamic localization precision analysis, see discussion above) and in the same range as the values of the previous STED‐FCS measurements.^[^ ^3^ ^]^ In contrast, trajectories with confined or free diffusion might have averaged over hopping events due to missing spatial or temporal resolution in those specific cases, artificially lowering the overall diffusion coefficient and expanding the confinement sizes (*L* > 400 nm) or might have simply expressed a completely different diffusion behavior, highlighting the heterogeneity of the plasma membrane.

Overall, our results highlighted that the Au NPs and especially the larger Ø40 nm Au NPs had an influence on the diffusion characteristics, both on small as well as long time scales and thus with respect to all diffusion coefficients (*D*_μ_, *D*_M_, and Γ) and also the confinement size *L*, although less in the latter case. Most importantly, despite the influence by the label, our ISCAT data congruently highlighted confined diffusion with a strong component of transient compartmentalization, where the confinement size ranged around 120–130 nm. With respect to this, we, in the case of the subset of trajectories that were best described by transient compartmentalized diffusion, could explore the confinement time *τ*_Conf_ (Equation 9) and the confinement strength *S*_Conf_ (Equation 10) metrics. While there was, for example, no significant difference (*p* > 0.05) between the confinement times for the different label sizes (<*τ*_Conf_> = 30 ± 30 ms for Ø20 nm Au NPs vs <*τ*_Conf_> = 24 ± 25 ms for Ø40 nm Au NPs), the confinement strength was significantly (*p* < 0.05) greater for lipids that were labeled with the larger Au NPs (<*S*_Conf_> = 4.5 ± 3.8 for Ø40 nm Au NPs vs <*S*_Conf_> = 2.7 ± 2.2 for Ø20 nm Au NPs). Given that there was, as discussed, no significant difference between the intracompartmental diffusion coefficient *D*_μ_ among lipids labeled with either size of the Au NPs, the significantly greater confinement strength of the larger Ø40 nm Au NP tagged lipids must have then primarily be manifested by the significantly slower intercompartmental diffusion coefficient *D*_M_ of lipids tagged with these larger Ø40 nm Au NPs (<*D*_M_> = 0.21 ± 0.14 μm^2^ s^−1^ for Ø40 nm Au NPs vs <*D*_M_> = 0.29 ± 0.18 μm^2^ s^−1^ for Ø20 nm Au NPs).

These two metrics thus suggested that the single trajectories that were best described as transient compartmentalized diffusion were, for both sizes of Au NPs, much more confined, both in terms of the transient confinement time *τ*_Conf_ and the confinement strength *S*_Conf_ than was apparent from the ensemble average analysis. In support of this interpretation, we note as before, from the same simple back of the envelope calculation, that the mean step length for free diffusion in two dimensions, (4 *D*_μ_ *t*_lag_)^0.5^, for as in our case *D*_μ_ ≈ 1 μm^2^ s^−1^ (Table 4) is *L*/2 ≈ 60–65 nm for *t* ≈ 2 *t*_lag_ ≈ 1 ms. This would mean that a lipid gold particle complex that originated at the center of a confining compartment, with size *L* = 120–130 nm, and that diffused exactly back and forth from the center to the confining barrier during an average confinement time of *τ*_Conf_ = 25 ms would have encountered the confining barrier a minimum of about 12 times before escaping to an adjacent compartment. This is considerably more times than what emerged from the ensemble average analysis, and with these many encounters with the confining barriers, even a very subtle difference in crossing the barrier to an adjacent compartment could have translated to a significant difference in confinement strength. However, given the large variability of the various parameter magnitudes reported here, this interpretation will need further confirmation. What is clear from this analysis is, however, that the subset of lipids experiencing transient compartmentalization was much more strongly confined, with longer confinement times, than what the results from the ensemble average analysis indicated.

### 2.6 Validation of Analysis Results by Comparison to Monte Carlo Simulated Diffusion on a Heterogeneous Lattice

We have, in a previous STED‐FCS work, shown the utility of comparing experimental data and Monte Carlo simulations that both revealed the presence of transient compartmentalized diffusion on a heterogeneous lattice.^[^ ^3^ ^]^ In this approach, we performed Monte Carlo simulations for diffusion in a continuous heterogeneous lattice with simulation parameters *P*_Hop_, for the probability that a molecule that encounters a confining barrier is able to hop between adjacent compartments, with an average compartment size *L*_S_, with and intracompartmental diffusion coefficient *D*_S_, and a localization precision *δ*_*xy*_^S^ (Supplementary Information).^[^ ^3^ ^]^ As shown previously,^[^ ^3^ ^]^ in these simulations, the parameter *P*_Hop_ is the main determinant of the confinement strength *S*_Conf_ = *D*_μ_/*D*_M_, where decreasing values suggest tighter confinement. Meanwhile, the simulation parameter *L*_S_ is the main determinant for the transition time between faster intra‐compartmental diffusion, and slower intercompartmental diffusion, while the simulation parameter *D*_S_ is the main determinant of the overall magnitude of the time‐dependent diffusion coefficient *D*(*t*). Significantly, with this approach, it is possible, via the simulated *P*_Hop_ parameter to go beyond the previous back of the envelope calculations for the minimum number of encounters that a diffusing lipid has with a confining barrier before escaping to an adjacent compartment, while also making it possible to evaluate the accuracy by which our analysis approach can extract the fit parameters for the confining compartment size, *L*, and the intracompartmental diffusion coefficient, *D*_μ_.

**Figure 4:**
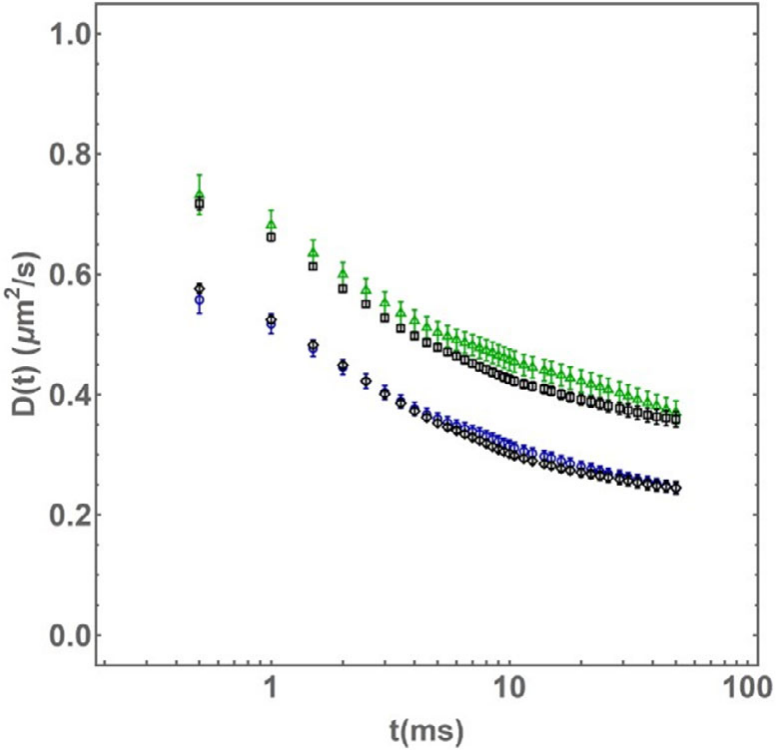
Comparison between experimental localization precision corrected time‐dependence of the ensemble average diffusion coefficient *D*(*t*) for lipids labeled with Ø20 nm (green triangles), and Ø40 nm (blue circles) gold particles, as well as respective most closely matching ensemble average Monte Carlo simulated data for each condition (black circles).

To approximate our experimental data to simulated data, we generated sets of 100 trajectories using the very same Monte Carlo simulations with gradually adjusted simulation parameters *P*_Hop_, *D*_S_, and *L*_S_, after which we applied simulated localization error to find conditions that most closely matched the shape of our experimental ensemble average *D*_app_(*t*) versus *t* curves (Figure 1c). We found that the experimental ensemble average ISCAT data for sAv‐Ø20 nm Au NP tagged DSPE could be well approximated by simulated data with the following parameters: *P*_Hop_ = 0.06, *D*_S_ = 1.1 μm^2^ s^−1^, *L*_S_ = 120 nm, and *δ*_*xy*_^S^ = 16 nm, while the data for sAv‐Ø40 nm Au NP tagged DSPE could be very well approximated by simulations with parameters *P*_Hop_ = 0.04, *D*_S_ = 0.8 μm^2^ s^−1^, *L*_S_ = 120 nm, and *δ*_*xy*_^S^ = 16 nm (**Figure** **4**).

We next applied our ensemble analysis pipeline as before to our simulated trajectories. This analysis showed that the matching simulated ensemble average data was also best described by the transient compartmentalized diffusion coefficient. The fit parameters for the analysis time interval of 0.5 ≤ *t* ≤ 50 ms, with the fit parameter of the corresponding experimental data in the same time range are shown in **Table** **5**. For completeness, the fit parameters for the other time ranges are reported, in Supplementary Figure S4, and Supplemental Table S7 and S8, Supporting Information. The very close resemblance of the curves in Figure 4, and of the extracted fit parameters in Table 5, between the experimental data and the simulated data confirmed that transient compartmentalized diffusion was a plausible interpretation for our experimental ensemble average data. These results further highlighted that the effect of the larger Au NPs was to slightly increase the confinement of lipids that were tagged with the larger Au NPs while also causing a slight decrease in the diffusion coefficient at all observation times.

**Table 5:** Comparison between experimental data and matching simulations.

Sample	*D*_μ_ (±S.E.) [μm^2^ s^−1^]	*D*_M_ (±S.E.) [μm^2^ s^−1^]	*L* (±S.E.) [nm]	*δ*_*xy*_ (±S.E.) [nm]	*τ*_Conf_ [ms]	*S*_Conf_
Ø20 nm Au/DSPEa)	0.87 ± 0.02	0.40 ± 0.00	110 ± 3.0	14.2 ± 0.2	7.5 ± 0.4	2.2 ± 0.1
*P*_Hop_ = 0.06 *D*_S_ = 1.1 μm^2^ s^−1^ *L*_S_ = 120 nm *δ*_*xy*_^S^ = 16 nmb)	0.86 ± 0.02	0.37 ± 0.00	106 ± 2.0	13.8 ± 0.2	7.6 ± 0.3	2.3 ± 0.1
Ø40 nm Au/DSPEa)	0.67 ± 0.02	0.26 ± 0.00	100 ± 2.3	13.5 ± 0.2	10 ± 0.6	2.6 ± 0.1
*P*_Hop_ = 0.04, *D*_S_ = 0.8 μm^2^ s^−1^, *L*_S_ = 120 nm, *δ*_*xy*_^S^ = 16 nmb)	0.71 ± 0.01	0.25 ± 0.00	99 ± 1.0	12.9 ± 0.1	9.9 ± 0.4	2.9 ± 0.1

a) Exact transient compartmentalized diffusion model (Equation 5.2) fit parameters of the ensemble average *D*_app_(*t*) curves of the experimental data, and the fit parameters obtained from the corresponding matching simulations.

b) Simulation parameters for matching simulations.

This comparison to the best‐matching simulated data further helped us with the interpretation of several additional aspects of our experimental data. First of all, from Table 5, it became apparent that our analysis approach following the direct fitting of the experimental *D*(*t*) dependencies resulted in an underestimation of the true value of the intracompartmental diffusion coefficient *D*_μ_, since the according values of the best matching simulated data, *D*_S_, were about 20% larger (Table 5). This is consistent with earlier suggestions by Kusumi et al., who recognized that a limited time resolution in sampling the molecular trajectories, which is always the case due to generic experimental delays in the signal detection and registration process, will always lead to an under‐estimation of mobility.^[^ ^4^ ^]^ In our experimental analysis pipeline, this underestimation was, however, minimized due to our dynamic handling of the localization precision and thus noise. Table 5 also highlights an underestimation of the values of both the compartment size, *L*, and the confinement strength metric, *S*_Conf_, which follows the argumentation given for the underestimation of *D*_μ_. Using the best matching simulated values of the hopping probability, *P*_Hop_ (Table 5), we can estimate that the diffusing lipids that were labeled with the smaller Ø20 nm Au NPs on average encountered a confining compartment barrier as many as 1/*P*_Hop_ = 1/0.06 ≈ 17 times, whereas a lipid labeled with the larger Ø40 nm Au NPs required on average 1/*P*_Hop_ = 1/0.04 ≈ 25 times before escaping. This is considerably more times than our earlier simplified back of the envelope calculations of 12 times from the direct fitting results, and follows the general underestimation. The results from the best matching simulations agreed with our interpretation from the direct fitting analysis of the ensemble average data in that the effect of the larger Ø40 nm Au NPs, as compared to the smaller Ø20 nm Au NPs, was to slow down the diffusion at all times *t* without altering the diffusion mode, and to make it slightly more difficult for the tagged lipids to escape the confining compartments. This same approach of comparing simulated data to experimental data could in principle also be performed at the single‐trajectory level although at current the absence of an analytical expression link between the experimental data and the simulated equivalent data would make this approach very computationally inefficient. What became clear, however, is that the back of the envelope calculations for the single‐trajectory analysis for the subset of trajectories best described as transient compartmentalized were clearly also an underestimate of the true transient confinement both in terms of the intracompartmental diffusion coefficient and the true hopping probability.

### 2.7 The Confinement Strength *S*_Conf_ Metric Enables a Direct Comparison of Diffusion Measurements in the Plasma Membrane from Different Techniques and Probe Types

In our final step, we aimed at comparing our experimental results to earlier related results in the literature. In the broader context of comparison of the present data with relevant literature, we found that the experimentally obtainable confinement strength *S*_Conf_ in unison with our simple Monte Carlo simulations for transient compartmentalized diffusion in a heterogeneous lattice was an effective metric for comparison of the diffusive motion in cellular membranes. A general difficulty was to adapt this framework to other methods used to explore diffusion dynamics of fluorescent phospholipid analogs on cell membranes, such as FCS, and its combination with super‐resolution STED microscopy (STED‐FCS). In this case, we defined *S*_Conf_ as the ratio of the diffusion coefficient at two distinct experimental conditions, which would represent a smaller and a larger observation domain. Therefore, we considered the diffusion coefficient calculated from data acquired at the highest available STED power, i.e., the smallest observation spot, as a measure of the intracompartmental diffusion coefficient *D*_μ_, whereas we defined the diffusion coefficient calculated from data acquired from the larger observation spot of the confocal recordings as a measure of the intercompartmental diffusion coefficient *D*_M_, i.e., *S*_Conf_ = *D*_μ_/*D*_M_ = *D*_STED_/*D*_Confocal_. We also estimated values of *S*_Conf_ from conventional FCS measurements of labeled fluorescent phospholipid analogs on living cell membrane, where the Arp2/3 specific inhibitor CK‐666 was used to disrupt the actin cytoskeleton, i.e., to remove the compartmentalization of the plasma membrane. In this case, diffusion measured in the presence of CK‐666 was effectively equivalent to the intracompartmental diffusion, that is, *D*_μ_, and diffusion of the same reporter on the unperturbed cell membrane was equivalent to *D*_M_. Thus, we defined the confinement strength, *S*_Conf_, for FCS in this case as *S*_Conf_ = *D*_μ_/*D*_M_ = *D*_Confocal_ (+CK666)/*D*_Confocal_ (−CK666). With this, our current ISCAT experiments and other previous SPT and SFMI data, we could gather values of *S*_Conf_ for multiple experiments of the diffusion of phospholipids (saturated DPPE and DSPE or unsaturated DOPE) in the plasma membrane of different living cells (Ptk2, IA32 MEF, NRK, T24 epithelial cells) using different labeling approaches (biotinylated lipid linked to avidin‐coated AU NPs (sAv‐AU) or a quantum dot (sAv‐QD655) with different PEG linkers (2000 or cap); and fluorescent lipid analogs with different dyes (Atto647N or Cy3) (for details see Supplementary Table S10, Supporting Information).

**Figure 5:**
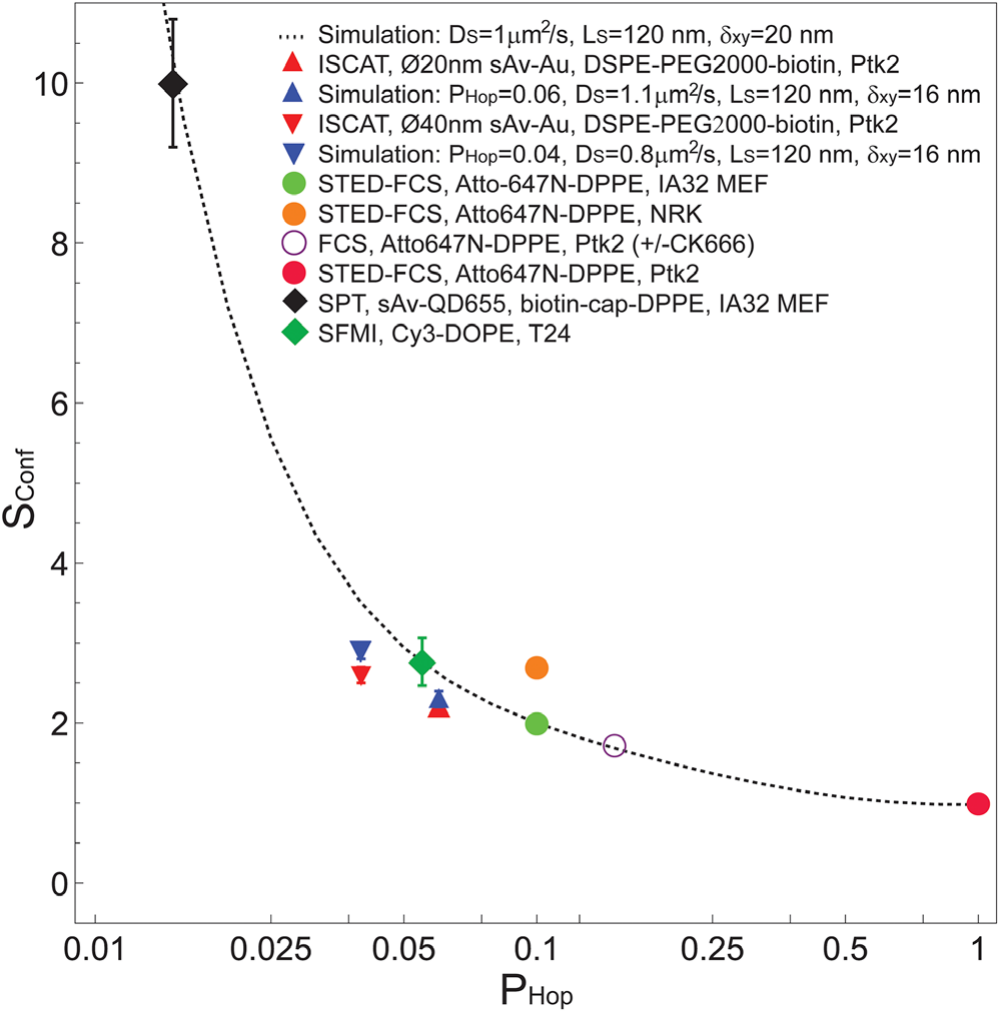
Comparison of ISCAT experimental results to our previous lateral diffusion in plasma membrane of live cells by STED‐FCS (closed circles), FCS (open circle), and SPT/SFMI (closed diamonds). To put the results from this study into context with previous related studies, we have plotted the confinement strength *S*_Conf_ = *D*_μ_/*D*_M_, as determined by comparative analysis of simulated trajectory data for compartmentalized diffusion, versus the hopping probability, *P*_Hop_, for our and others’ experimental data along with simulated data as indicated in the Figure legend and in detailed described in Supplementary Table S10, Supporting Information. The blue triangles highlight the values from the best matching simulation to the current ISCAT data (using the parameter values stated in the legend), and the dotted line connects value pairs from simulations with different input parameters using parameter values of *D*_S_ = 1.0 μm^2^ s^−1^, *L*_S_ = 120 nm, *δ*_*xy*_^S^ = 20 nm, and different hopping probabilities *P*_Hop_ (dotted line).

For a better comparison of the different data, we added matching values of the hopping probability *P*_Hop_ to each *S*_Conf_ value. For some data (our current ISCAT data as well as the previous STED‐FCS data) values of *P*_Hop_ were present from best matching simulations (using either the here or previously^[^ ^3^, ^22^ ^]^ introduced simulations). For all the other experimental data, we first generated a trend line obtained by fitting a fourth order polynomial to the ensemble average analysis results of Monte Carlo‐simulated transient compartmentalized diffusion in a heterogeneous lattice with simulation parameters of *D*_S_ = 1.0 μm^2^ s^−1^, *L*_S_ = 120 nm, *δ*_*xy*_^S^ = 20 nm that closely resembled the relevant experimental data at a range of hopping probabilities, *P*_Hop_, of 1, 0.5, 0.25, 0.1, 0.05, and 0.025 (Supplementary Figure S5, and Supplemental Table S9, Supporting Information). These simulations enabled us to obtain an estimate of the relationship between the experimentally determinable confinement strength metric *S*_Conf_ and the arguably much more informative hopping probability *P*_Hop_. We subsequently used this trendline to estimate the hopping probabilities *P*_Hop_ for related experimental data for which the hopping probability was not available, and with this, we could plot all the different value pairs (*S*_Conf_, *P*_Hop_), as given in **Figure** **5**.

From this comparison, it became apparent that there is a subset of measurements in the literature, from experimental methods that are able to sample at a localization precision of <50 nm, and a temporal resolution of <1 ms,[^1^, ^2^, ^8^] that reach an approximate consensus on the values of *S*_Conf_ and *P*_Hop_ and with that on the value for the intracompartmental diffusion coefficient of lipids of about 0.5–1 μm^2^ s^−1^ in the plasma membrane of mammalian cells. Further, the data in Figure 5 reconfirmed that our current analysis approach on the ISCAT data correctly identified stronger transient compartmentalized diffusion with a *P*_Hop_ ≤ 0.25 but that very weak transient compartmentalized diffusion with *P*_Hop_ ≥ 0.5 might be misinterpreted as free diffusion, but with a slower diffusion coefficient than the corresponding simulated diffusion coefficient *D*_S_. The simulated data as well as trend line furthermore re‐confirmed that the value pairs generated from our experimental ISCAT data acquired at 2 kHz underestimated *S*_Conf_ and thus the true intracompartmental diffusion coefficient *D*_μ_ whereby the discrepancy increased with decreasing values of *P*_Hop_, i.e., increased compartmentalization strength.

Overall, with the trend line and all the comparing experimental value pairs in hand, the ISCAT data presented in this work and the related data from earlier studies STED‐FCS, FCS, and SPT studies with a range of probes, from potentially more invasive highly multivalent streptavidin QDs to less invasive dye labels (Atto647N, Cy3), started to coalesce to a unifying picture for the diffusion of lipids in the plasma membrane of mammalian cells. In this picture, lipids were on average transiently confined at a spatial scale of about 100 nm for about 5–10 ms where the short‐range, unhindered, diffusion coefficient *D*_μ_ is on the order of 1 μm^2^ s^−1^, while the long‐range, weakly hindered diffusion coefficient, with minimally invasive probes, *D*_M_ was approximately twofold smaller (*S*_Conf_ ≈ 2) corresponding to a hopping probability, *P*_Hop_, on the order of 1/20–1/10 (0.05–0.1). This comparison of Figure 5 furthermore suggested that our labeling approach in this study, of using comparatively large sAv‐Ø20 nm and sAv‐Ø40 nm diameter Au NPs, to track lipid diffusion in the plasma membrane, did actually not significantly alter the observed diffusion rates as very similar diffusion rate magnitudes were observed within a related single‐molecule tracking study with a minimally invasive Cy3 dye labeled lipid analog in T24 cells,^[^ ^2^ ^]^ and by STED‐FCS with a minimally invasive Atto647N dye‐labeled analog in IA32 MEF and NRK cells.^[^ ^3^ ^]^

## 3 Conclusion

In this work, we have employed interferometric scattering (ISCAT) microscopy in combination with an unbiased analysis pipeline to re‐visit the diffusion dynamics of a lipid analog (DSPE‐PEG2000‐biotin), labeled with either sAv‐Ø20, or sAv‐Ø40 nm diameter Au NPs in the plasma membrane of live Ptk2 cells, at an acquisition rate of 2 kHz and a localization precision of <15 nm. Our unbiased analysis suggests that the diffusion dynamics in the plasma membrane (and by inference the under‐lying plasma membrane nano‐organization) in Ptk2 cells is complex and heterogeneous. This analysis further showed that the most likely diffusion modes for our DSPE lipid analog, both at the ensemble average, and at the single‐trajectory level, were independent of the size of the sAv‐Au NPs. This result suggests that we were sampling the same underlying plasma membrane nano‐organization with both the smaller sAv‐Ø20 nm Au NPs, and the eightfold by volume larger and potentially more invasive sAv‐Ø40 nm Au NPs.

At the ensemble average level, our analysis showed that the most likely diffusion mode, for lipids, that had been post‐labelled with either smaller Ø20 nm, or larger Ø40 nm diameter Au NPs, was transient compartmentalized diffusion (Equation 6.2) with an average compartment size *L* ≈ 100–110 nm. The average diffusion coefficient within the compartments, the intra‐compartmental diffusion coefficient, was *D*_μ_ ≈ 0.9 μm^2^ s^−1^ for lipids labeled with the smaller Ø20 nm Au NPs, respectively *D*_μ_ ≈ 0.7 μm^2^ s^−1^ for the larger Ø40 nm Au NPs, while the average long‐range, intercompartmental diffusion coefficient, was *D*_M_ ≈ 0.4 μm^2^ s^−1^ for lipids labeled with the smaller Ø20 nm Au NPs, respectively *D*_M_ ≈ 0.3 μm^2^ s^−1^ for lipids labeled with the larger Ø40 nm Au NPs (Figure 1d and 2, and Supplementary Table S3–S6, Supporting Information). The average transient confinement time, confinement strength, and hopping probability on encountering the confining barriers of lipids labeled with the smaller Ø20 nm Au NPs were *τ*_Conf_ ≈ 7.5 ms, *S*_Conf_ ≈ 2.2, and *P*_Hop_ ≈ 0.06, respectively, *τ*_Conf_ ≈ 10 ms, *S*_Conf_ ≈ 2.6, and *P*_Hop_ ≈ 0.04 for the larger Ø40 nm Au NPs. Collectively, these results confirmed that the larger sAv‐Ø40 nm Au NPs were more invasive as this larger probe induced a reduction in the lipid diffusion rates, and tighter transient lipid confinement compared to lipids that were labeled with the smaller sAv‐Ø20 nm Au NPs. Significantly, our use of complementary Monte Carlo simulations enabled us to validate our analysis results and, via the simulation parameter of the hopping probability *P*_Hop_, provided a clear link to the number of encounters with a confining barrier that would be required before escaping to an adjacent compartment. This approach validated that our experimental data could be explained as transient compartmentalized diffusion, but it also suggested that our extracted value of the intracompartmental diffusion coefficient *D*_μ_, was likely about 20% smaller than the actual true value, and that this underestimate also had a similar effect on the extracted values for the compartment size *L*, and the confinement strength metric, *S*_Conf_.

Neither of the Au NPs that was used in this work is the ideal probe in terms of potential invasiveness due to the size, and potential for artificial Au‐probe‐induced cross‐linking. The effect of probe size was, in this case, thought to be minimal as a consequence of the prevailing Saffman–Delbruck formula for diffusion in a membrane, since the viscosity of the aqueous medium into which the Au NP extended was much lower as compared to the much higher viscosity within the membrane where the artificial lipid resided.^[^ ^33^ ^]^ Probe‐induced artificial cross‐linking, both because of the tetravalency of streptavidin, and because there were likely multiple streptavidin per Au NP, was a much bigger issue. While we showed that we could track single Au NPs at fast 2 kHz acquisition rates with high precision, we could not exactly know how many lipids each Au NP was bound to due to possible crosslinking effects caused by the probes. Yet, our approach to compare lateral diffusion data from related techniques (Figure 5), based on the experimentally accessible confinement strength metric *S*_Conf_, and the hopping probability *P*_Hop_ parameter determined from simulations, suggested that labeling using comparatively large sAv‐Ø20 nm and sAv‐Ø40 nm diameter Au NPs to track lipid diffusion in the plasma membrane, did not significantly alter the observed diffusion rates. In fact, very similar diffusion rate magnitudes were observed in a related single‐molecule tracking study with a minimally invasive Cy3 dye labelled lipid analog in T24 cells,^[^ ^2^ ^]^ and by STED‐FCS with a minimally invasive Atto647N dye labelled analog in IA32 MEF and NRK cells.^[^ ^3^ ^]^ This comparison, which spans a variety of cell lines as well as very different experimental techniques and conditions, hints at an increasingly unifying picture for the ensemble average diffusion of lipids in the plasma membrane that is consistent with transient compartmentalized diffusion with a hopping probability of *P*_Hop_ ≈ 0.05–0.1, a confinement strength of *S*_Conf_ ≈ 2–3, a compartment size of *L* ≈ 100 nm, a confinement time of *τ*_Conf_ ≈ 5–10 ms, an intercompartmental diffusion coefficient of *D*_μ_ ≈ 1 μm^2^ s^−1^, and an intra‐compartment diffusion coefficient *D*_M_ that is twofold to threefold slower. This unifying picture on the ensemble average diffusion in plasma membranes is consistent with the hydrodynamic model proposed by Oppenheimer and Diamant, for diffusion on a membrane with immobile obstacles, when considering a fraction of occupied area typical for cell membranes.^[^ ^34^ ^]^ Previous work has further pinpointed the cortical actin cytoskeleton as a barrier for long‐range diffusion at spatiotemporal scales that are consistent with our unifying picture.^[^ ^3^ ^]^

The analysis results at the single‐trajectory level were further indicative of a much more complicated nano‐organization of the plasma membrane with as per our analysis four distinct layers of organization. 1) Areas with no boundaries, as indicated by the observation of freely diffusing lipids (Equation 2). 2) Areas with adjacent smaller compartments with a size of about 120–130 nm and with fairly regularly time transitions every 25–30 ms between adjacent compartments, as indicated by the transient compartmentalized lipid diffusion (Equation 6.2). 3) Areas with larger compartments with a size of about 400–500 nm and transition times that are longer than the truncated analysis time of >100 ms, as indicated by the confined lipid diffusion (Equation 5.2). 4) Areas that would be consistent with adjacent compartments, with a broad range of sizes, and a broad range of transition times, as indicated by the anomalous lipid diffusion (Equation 7). These data were, however, due to the large observed heterogeneity broadly distributed such that further validation is required in future work. In particular, it would be fruitful to apply our unbiased single‐trajectory analysis approach to systematically explore differences in diffusive behavior in the plasma membrane at the single‐trajectory level in distinct subcellular local parts of the cell such as the lamella as compared to lamellipodium as the underlying subcellular organization of the cortical actin cytoskeleton is known to be distinctly different in these parts.^[^ ^35^ ^]^ Furthermore, the same approach could be used to systematically explore relative differences in the diffusive behavior at the single‐trajectory level in response to various biochemical stimuli that are known to affect the plasma membrane nano‐organization or the cortical actin cytoskeleton.

In summary, our presented results suggest that there is a collection of results in the literature that supports the observation of transient compartmentalized diffusion of lipids in the plasma membrane at the ensemble average level with similar diffusion parameters, but that the diffusive motion of lipids and by extension the nano‐organization of the plasma membrane is much more heterogeneous than can be fully resolved just from standard ensemble average analysis. This justifies our approach to extend the analysis by including single‐trajectory analysis, as well as forgoing the selection of a single diffusion mode in favor of finding out the most likely one from a selection of several diffusion modes. Coincidentally, this approach also seeks to fully exploit the potential of SPT, in which diffusion heterogeneities at the single‐molecule level can be observed and studied.

## Supporting Information

The authors have cited additional references within the Supporting Information.^[^ ^36^, ^37^, ^38^, ^39^, ^40^, ^41^ ^]^

## Conflict of Interest

The authors declare no conflict of interest.

## Supporting information

Supplementary Material

## Data Availability

The data that support the findings of this study are available from the corresponding author upon reasonable request.
