# Supplementary material for: High‐Speed Interferometric Scattering Tracking Microscopy of Compartmentalized Lipid Diffusion in Living Cells
Source: Chemphyschem. 2025 Sep 28;26(21):e202400407. doi: 10.1002/cphc.202400407 (PMC12597221; doi:10.1002/cphc.202400407)
Supplement: Supplementary file 1 — Supplementary Material [file CPHC-26-e202400407-s001.zip › Supporting Information.pdf]

SUPPLEMENTARY INFORMATION

High-speed Interferometric Scattering Tracking Microscopy of compartmentalized lipid diffusion in living cells

Francesco Reina, Christian Eggeling, B. Christoffer Lagerholm

Material and Methods

ISCAT MICROSCOPE

ISCAT experiments were performed on a custom built, following the protocol in [36], that has been previously described [15] with some useful modifications. The output from a 660nm solid-state laser diode (OdicForce) was scanned in two directions (equivalent to the x and y on the sample plane) by two acousto-optic deflectors (AOD, Gooch & Housego and AA Opto-Electronics). The scanned output was then linearly polarized, relayed to the back focal plane of the objective via a two-lens telecentric system, passed through a polarizing beam splitter and circularly polarized by a quarter wave plate (B.Halle). The light was finally focused by a Plan Apochromatic 60x, 1.42NA oil immersion objective (Olympus), mounted in an inverted geometry. As stated in the introduction, the reflected component by the glass-sample interface and the back-scattered component by the sample were collected by the same objective, and reflected onto the detection path by a polarizing beam-splitter. The final image is obtained by focusing these two interfering beams onto the CMOS camera sensor (Photonfocus MV-D1024-160-CL-8) to acquire time lapses with an effective magnification of 333x (31.8nm effective pixel size).

In addition to this imaging mode, the microscope was also equipped with a total internal reflection fluorescence-capable channel. A 462nm wavelength solid state laser diode output was focused on the back aperture of the objective. TIR illumination condition was achieved via a movable mirror, until the reflection of the illumination beam was visible on the other side of the back-aperture. The fluorescence signal was separated from this reflection by means of an appropriately placed dichroic mirror, and imaged onto a difference CMOS camera (PointGrey Grasshopper 3). The labelling of cells with a fluorescent lipid analogue ensured that the sample could be correctly identified in a second, independent way. However, this part of the setup was not optimized to perform fluorescence imaging experiments, and it was used merely as a guide for the user.

Stabilization of the imaging plane was achieved by a piezo-actuated objective positioner (PiezoSystem Jena) in open-loop configuration. This ensured enough stability in the focus to perform the intended measurements. A summarizing scheme for the imaging setup is given in Supplemental Figure S1.

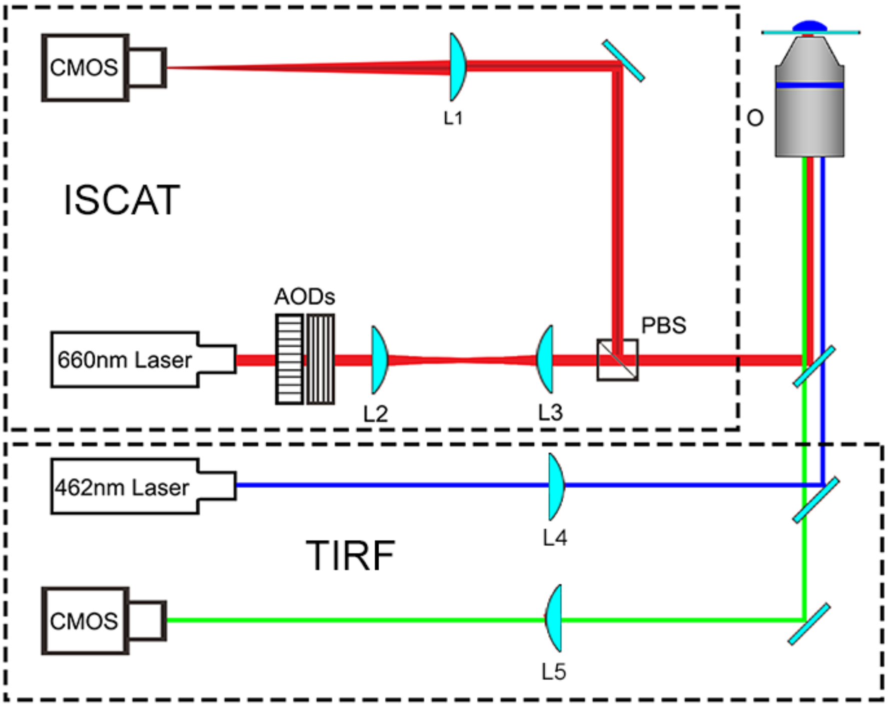

Supplemental Figure S1. Basic scheme of the ISCAT microscopy setup employed for the experiments**.** The beam from a 660nm laser diode is scanned in the x and y directions by two Acousto-Optic deflectors (AODs, Gooch & Housego and AA Opto-Electronics). The scanning is then relayed by a telecentric system (L2, L3) to the back focal plane of the objective (O, Olympus PlanApo, 60x 1.42 NA oil immersion), mounted in inverted geometry. The component of the incident light backscattered by the sample and the reflection of the beam caused by the glass-sample interface are collected by the same objective. The polarization of the beam is adjusted so that the returning beams are then reflected by a Polarizing Beam Splitter (PBS) on the imaging camera (CMOS, Photon Focus MV-D1024-160-CL-8). The ISCAT image is formed on the camera using a 1000mm tube lens (L1, Thorlabs), so that the effective pixel size on the sensor is 31.8nm. Simultaneous fluorescence imaging was achieved by focusing the light (L4) from a 462nm laser diode in TIRF Mode with the use of a moving mirror. The fluorescence signal was detected by a second CMOS camera (FLIR Machine Vision, Grasshopper 3), after focusing with a 500mm lens (L5).

ISCAT IMAGE PRE-PROCESSING

Particle detection in ISCAT microscopy is hindered by the presence of spurious reflections in the optical setup [15, 17b, 19]. We therefore applied a temporal median filter to the final image. Briefly, a stack of one hundred images is collected while the sample is moved by the user. If the operation is performed correctly, i.e., the sample is displaced enough while still being in focus, the median filter obtained from this image stack therefore will contain only the fixed contribution to the signal [37]. By using this image as background signal, it is possible to dramatically improve the contrast of the sample in the final detected time-lapses. However, the aforementioned median filtering operation is seldom not sufficient to obtain enough particle contrast to ascertain the position of the particle. This is especially true in the case of samples with high scattering backgrounds such as cells. For this reason, we applied a second background reduction strategy, where we subtract the average intensity projection of the detected, median-filtered time lapse. In this way, the high-scattering cell landscape is mostly filtered out, facilitating the detection of the target gold nanoparticles (Supplementary Figure S2).

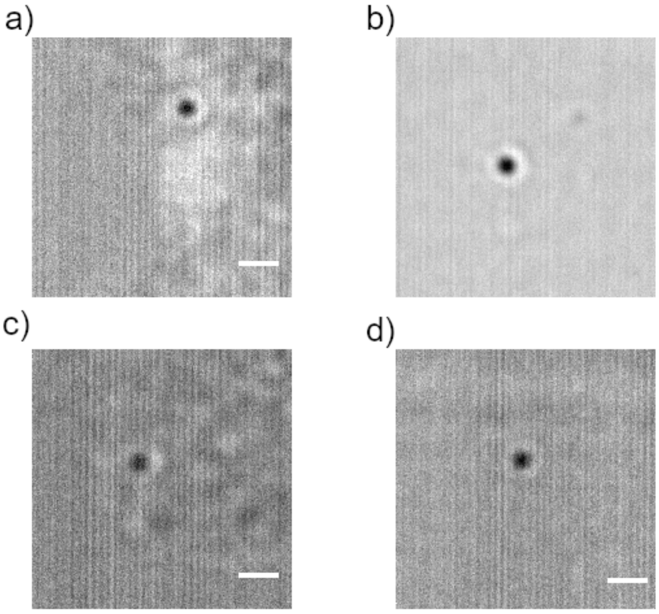

Supplemental Figure S2. Evaluation of scattering contrast of the gold nanoparticle tags in different environment. a) and b): Streptavidin-coated 40nm-diameter gold nanoparticle, respectively, on a PTK2 cell surface and on immobilized on a glass substrate. c) and d): Streptavidin-coated 20nm-diameter gold nanoparticle, respectively, on a PTK2 cell surface and on immobilized on a glass substrate. On the ISCAT images detected on cell surfaces, we applied a median filter and temporal average to enhance the contrast. On the nanoparticles immobilized on glass, only a median filter was applied.

LIPIDS AND CELL LINE

Atto488-labelled DOPE (1,2-Dioleoyl-*sn*-glycero-3-phosphoethanolamine) was purchased from Atto-Tec. DSPE-PEG(2000)-Biotin (DSPE: 1,2-distearoyl-sn-glycero-3-phosphoethanolamine, 2kDa PEG linker between the phospholipid polar head and the biotin)**,** henceforth referred to simply as DSPE-PEG-Biotin, was purchased from Avanti Polar Lipids. Lipid stock solutions were stored at -20C in chloroform. Gold Nanoparticles of 20 nm and 40 nm diameter **(Ø)**, streptavidin coated, where purchased from BBI solution in stocks, the concentration of which is expressed as 10 OD (optical density). Cell experiments were performed in PtK2 cells derived from rat kangaroo (Potorus tridactylis) kidney tissue. These were cultured following standard protocols, growing them in Dulbecco Modified Eagle Serum (DMEM, Sigma Aldrich), supplemented with ~15% FBS (Fetal Bovine Serum), L-Glutamine, and Penicillin-Streptomycin.

CELL MEMBRANE LABELLING

PtK2 cells were seeded and left to proliferate on methylated-spirits cleaned glass supports (25mm diameter, #1.5 thickness coverslips), and used at a stage where they did not yet reach confluency. A sufficient separation between the cells is deemed necessary to ensure that the membrane of each cell was not affected by the presence of neighbouring cells that may cause deformation. This translates to an estimated 50-70% confluency. Before the labelling, to allow a more comfortable and secure application of the labelling protocol, the glass supports were mounted in a water-tight steel chamber (Attofluor chambers, Thermo Scientific). The cell labelling procedure was adapted from the protocol described in [38]. A stock solution of DSPE-PEG-Biotin in 1:1 Chloroform-Methanol at 10 mg/ml was desiccated via nitrogen gas flow, and the lipid suspended again in absolute ethanol to a concentration of 20 mg/ml. This was diluted in L15 medium to a final concentration of 0.2 mg/ml, and incubated at 37°C for 20-30 minutes. In the same buffer, a small concentration of Atto488-DOPE was dissolved, in order to facilitate detection of the labelled cells by using the fluorescent channel of the ISCAT microscope. After the incubation with the biotinylated lipids, the cells were washed with fresh L15 buffer, and incubated for 10-15 minutes at 37C with a solution of 0.6 µM of streptavidin-coated Ø20nm or Ø40 nm diameter (Ø) gold nanoparticles in L15 buffer. Afterwards, the cells were once again rinsed with fresh L15 buffer, and used for the experiments. This protocol produced a sparse labelling of cells (~1-2 nanoparticles per cell, with multiple labelled cells).

IMAGING CONDITIONS

The glass support with the cells was positioned on the microscope stage while still inside the steel Attofluor chamber used for labelling. The cells were imaged in L15 medium at a temperature of 37°C, in room atmosphere and humidity, with a temperature control system (Warner Instruments). The laser power area density used to illuminate the cells for ISCAT imaging was 17.5 kW/cm^2, at an illumination wavelength of 660nm. It has been shown in similar cell lines that prolonged exposures to even higher power densities, at the same wavelength as our experiments, are well tolerated [39]. Using the CMOS camera, we collected 2000 frames long movies in a 200x200 px^2 region, corresponding to roughly 41μm^2 imaging field of view, with 0.227 ms exposure time, and an image acquisition rate of 2kHz sampling rate. While related experiments have been carried out at much faster sampling rates, it has been shown that similar sets of parameters are sufficient to describe the scenario herein considered [3].

TRAJECTORY DETECTION

Single Particle Tracking data analysis requires the trajectories of the particles to be extracted from the collected movies. The movies were collected in TDMS file format with a LabView software (courtesy of the Kukura Laboratory, University of Oxford), and converted to TIFF image stacks with a home-written MatLab code, based on the ConvertTDMS function by Brad Humphreys (https://www.github.com/humphreysb/ConvertTDMS, last retrieved March 23, 2020). Before particle tracking, all the movies were elaborated by subtracting the median filter, as previously described [15]. In addition to this, the average intensity projection of the movie was obtained and subtracted from every movie frame, in order to separate the moving fraction of the sample from the static background (Supplemental Figure S2). Image processing was performed using the FIJI platform [60]. Tracking was performed using the Spot Detection function in Imaris 9.5 (Bitplane, Oxford Instruments). Subsequent trajectories were imported in text format for post-processing in Mathematica (version 12.0.0.0; Wolfram Research) with custom written codes.

ANALYSIS OF SINGLE PARTICLE TRAJECTORIES

In order to thoroughly analyse and extract correct information from the collected data, we have refined the data analysis protocol previously introduced in [22]. The distinguishing features of this analysis protocol is the treatment of the localization uncertainty and the implementation of a robust, data-driven statistical method of single trajectory classification based on the most likely model of diffusive motion from a set thereof. Furthermore, in order to form a robust analysis pipeline with constant statistical sampling of measured displacements, we have restricted our analysis to single trajectories that contained at least 500 consecutive localizations, thus corresponding to a trajectory duration of 250 ms at our sampling rate of 2kHz. Furthermore, we truncated all trajectories into segments of 500 consecutive localizations.

In our analysis, we calculated the Mean Squared Displacements (MSD), for each single trajectory from

$$MSD\ (\mathrm{n\ t}_{lag}) = \frac{1}{N-n}\sum_{i=1}^{N-n}(r_{i+n}-r_i)^2 \qquad (Eq.S1)$$

where r_i is the particle position at time t_i, for all available displacements at a given time lag interval t = n t_{lag}, where t_{lag} is the interval between two successive observations of the same particle, in this case 0.5 ms, resulting from a sampling frequency, 1/ t_{lag}, of 2kHz. In the text, we refer to “single trajectory analysis” when the data arising from each single trajectory is analysed, and to “ensemble average” analysis when the MSD values of the trajectories belonging to the same dataset are averaged and then analysed.

The conventional data analysis strategy is to subsequently fit the experimentally determined MSD(n t_{lag}) dependence for a particular analysis time range, $t_{start} \le t \le t_{stop}$ to a relevant model of diffusive motion such as the expression Brownian (free) diffusion on a two dimensional plane, i.e.

$$\mathrm{MSD}(\mathrm{n\ t}_{lag}) = 4\,\mathrm{D\ n\ t}_{lag}\left(1-\frac{2\mathrm{R}}{\mathrm{n}}\right) + 4\,\delta_{xy}{}^2 \qquad (Eq.S2)$$

where we have also included two factors that are necessary for experimental MSD data. These correction factors are: 1) an additional correction factor, (1-2R/n), to account for motion blur as a consequence of particle motion during the camera integration time [25], and 2) a constant term, 4 δ_{xy}^2, to account for the dynamic localization uncertainty by which each particle position can be determined [26] with an assumption that that the dynamic localization precision in the x-direction, δ_x, and y-direction, δ_y, are equal, i.e. $\delta_x = \delta_y = \delta_{xy}$. The motion blur correction term, which primarily affects the first few data points n, depends on the mode of illumination of the sample. In the case of full frame averaging, with exposure time t_{exp} = 0.227 ms, as employed here, R=1/6 (t_{exp}/ t_{lag}) = 1/6 (0.227 ms/ 0.5 ms) [12], [15], [16]. The localization error term, $\delta_{xy}{}^2$ which contributes a constant y-offset, primarily depends on the signal-to-noise of the microscope set-up where $\delta_{xy}{}^2$ is the dynamic localization uncertainty. These correction factors are an absolute requirement for fast sampling rates as these factors make the trajectories highly non-linear even when simple free diffusion is involved.

While Eq. S2 is suitable for quantitative analysis, we find it much more informative, due of the complexities that are introduced by the above correction terms, to instead transform the MSD(n t_{lag}) data to the same units of [length^2/time] as the diffusion coefficient for our case of 2D diffusive motion on a plane as.

$$D_{app}(\mathrm{n\ t}_{lag}) = \frac{MSD(\mathrm{n\ t}_{lag})}{4\,\mathrm{n\ t}_{lag}\left(1-\frac{2R}{n}\right)} \qquad (Eq.S3)$$

The right-hand side of Eq. S3 can be thought of as an apparent diffusion coefficient, D_{app}(n t_{lag}) from which it is now possible to directly qualitatively evaluate the time dependence of the diffusive process from the trajectory data even in the absence of curve fitting to any specific model.

The data, transformed through Eq. S3, was subsequently analysed by least squares non-linear curve fitting, weighed to a range of theoretical models for diffusive motion, all of which directly incorporated the camera blur corrected effect of localization noise as shown in Eq. S2. The generic relationship for the data models that we used for this analysis is obtained by substituting Eq. S2 in Eq. S3:

$$D_{app}(\mathrm{n\ t}_{lag}) = D(\mathrm{n\ t}_{lag}) + \frac{\delta_{xy}{}^2}{\mathrm{n\ t}_{lag}\left(1-\frac{2R}{n}\right)} \qquad (Eq.S4)$$

where we have split the calculated apparent diffusion coefficient D_{app}(n t_{lag}) into its two distinct contributions: 1) the diffusive process D(n t_{lag}), and 2) an artificial diffusion-like camera blur-corrected localization error term, in which δ_{xy} is the localization uncertainty. Eq. S4 is equivalent to Eq 2 in the main text where t=n t_{lag}. The diffusion component D(n t_{lag}) in Eqs. 2 and S4 can take on a potentially

unlimited number of different expressions to describe a range of theoretical diffusive motion models. In this work we have considered a total of six plausible models for the diffusion component. These expressions are shown in Table 2 in the main text. By substituting the expressions of D(n t_{lag}) shown in Table 2, corresponding to different plausible diffusive motion models in Eq. 2, we obtain a set of functions to fit to each of the both the ensemble average and the single trajectory experimental D_{app} curves.

Model fits were performed using a nonlinear least-squares fitting routine Mathematica (version 12.0.0.0; Wolfram Research) using the NonlinearModelFit[] command, where the data points were weighted according to the magnitude of the inverse of the variance of the D_{app}(n t_{lag}), and with a fitting confidence level of 0.99. For these fits, we sampled the data points non-linearly, in order to ensure that the points at larger time lags are not overwhelmingly weighted compared to the fewer points at the earliest time lags. This is done as the first few time points are more informative on short-lived events, such as transient confinements. The sampling was operated by converting the time axis to a logarithmic scale, and sampling in intervals of length $(\log_{10} T - \log_{10} t_0)/(0.5 * T/t_0)$, where T is the maximum time range considered for the analysis. We have chosen to perform our analysis at six different time ranges, that is, six different values of T (5 ms, 10 ms, 25 ms, 50 ms, 75 ms and 100 ms), in order to cover a variety of time regimes with our study. With our data sampling approach, for example, for T=50ms (corresponding to n=100 data points), we selected time points that were spaced (Log[50ms]-Log[0.5ms])/75 apart. After rounding to the closest values and removal of duplicate time points, this results in 46 roughly evenly spaced time points on a log scale where n={1, 2, 3, 4, 5, 6, 7, 8, 9, 10, 11, 12, 13, 14, 15, 16, 17, 18, 19, 20, 22, 23, 24, 26, 28, 29, 31, 33, 35, 37, 40, 42, 45, 48, 51, 54, 58, 61, 65, 69, 74, 78, 83, 88, 94, 100} that we used for the fitting. This effectively serves to place extra emphasis on the initial time points, where we observe the greatest change in magnitude of the apparent diffusion coefficient, due to the influence of the localization error and due to the transient confinements of the particle trajectories. All the analysis routines herein described, can be easily reconstructed using the Python package reported in [23].

VIBRATION CORRECTION OF EXPERIMENTAL DATA

Our experimental data suffered from slight environmental vibration artefact with a frequency corresponding roughly to that of a common fan frequency of about 8,500 rpm (≈142 Hz). This was most clearly visible in the control trajectory data of immobile gold particles on glass (Supplemental Figure S3). Prior to the quantitative analysis, we thus removed the influence of these environmental vibrations by median filtering in Fourier space the frequencies ranging from 140-160 Hz from the MSD curves of each single particle trajectory. A comparison between the original and the vibration corrected data is shown in Supplemental Figure S3, and the parameters for corrections are given in Supplemental Table S1.

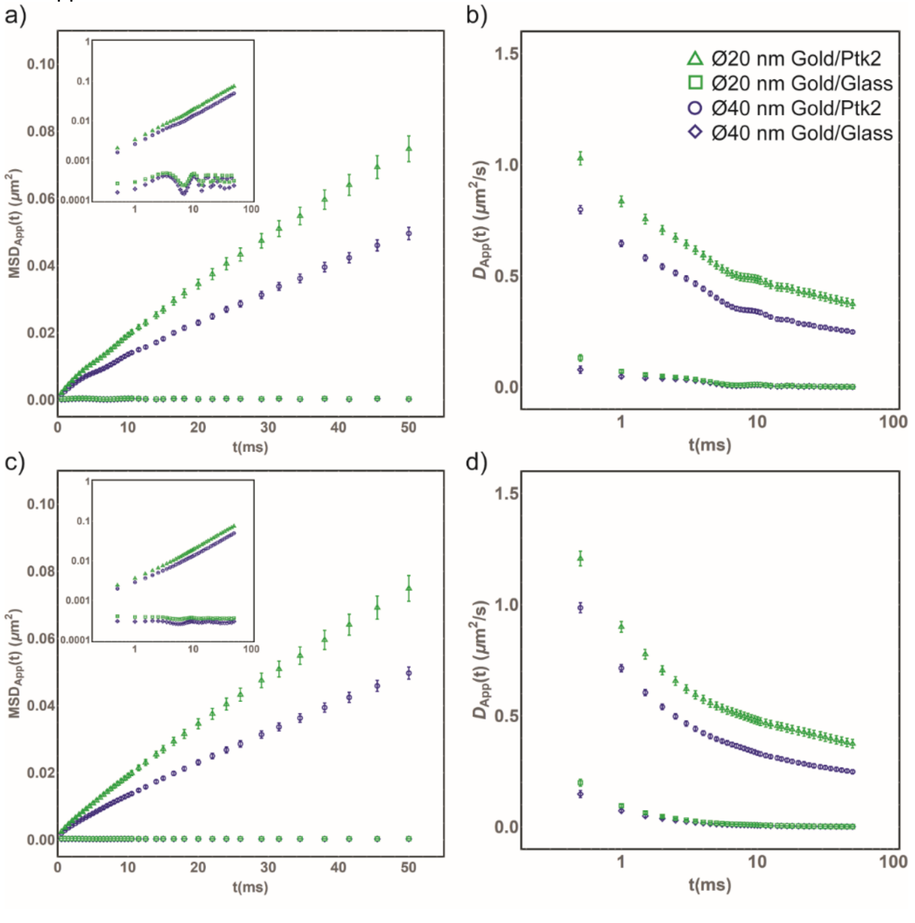

Supplemental Figure S3. Comparison between vibration corrected and original Mean Squared Displacement and Apparent Diffusion Coefficient curves**.** a) Ensemble average of all experimental Mean Squared Displacement curves obtained from the experimental trajectories, before vibration correction. In the insert, the same data but in a log-log representation, to further highlight the presence of environmental vibrations. b) Ensemble average of all Apparent Diffusion curves obtained from the experimental trajectories, before vibration correction. c) Same data as in a), after median filtering out in Fourier space out the frequencies in Supplemental Table S1. In the insert, the same data but in a log-log representation. d) Same data as in b), after median filtering out in Fourier space the frequencies in Supplemental Table S1

Table S1. Vibration correction summary statistics

Sample	Frequency (±st.d.) (Hz)	Frequency (±st.d.) (rpm)	RMS (±st.d.) (nm)
DSPE -PEG2000-biotin / sAv-Au (Ø20nm) / Ptk2	143±3	8600±200	7.8 ± 2.7
DSPE-PEG2000-Biotin / sAv-Au (Ø40nm) / Ptk2	143±3	8600±200	7.5 ± 3.1
sAv-Au (Ø20nm) / Glass	143±3	8600±150	4.2 ± 1.4
sAv-Au (Ø40nm) / Glass	143±2	8600±130	4.4 ± 1.4

STATISTICAL EVALUATION OF THE MOST LIKELY DIFFUSION MODEL

In this work, we have chosen to adopt a data-driven diffusion motion classification that does neither require *a priori* knowledge nor assumptions on which diffusive motion model to assign to a particular data set. This approach is a refined extension to our previous work [22], with refinements aimed at increasing the robustness of the approach in order to extend the applicability to the analysis of single trajectories. The challenge of analysing single trajectory data in this context stems from the fact that single trajectories are in general much noisier than the ensemble average of each dataset's trajectories, as the averaging step involves significantly fewer displacements.

In this extended approach, we determined the most likely diffusion model, from the set of plausible models (Eqs. 2-7), by calculating the Bayesian Information Criterion (BIC) from the results for each data model, according to the formula:

$$BIC(model) = n \ln\left({RSS}/{n}\right) + k\, ln(n) \qquad (Eq. S5)$$

where n is the number of data points used for the fitting, RSS is the residual sum of squares, and k is the number of free parameters in the fit. The first term in Eq. S5 is a measure of the Goodness of Fit while the second term adds a penalty function that scales linearly with the addition of each free parameter to the model. Thus, while normally a model with a larger k would inevitably result in a fit with a smaller RSS, the use of BIC as a classification metric allows to evaluate the models more equitably. A smaller BIC value equates to better fit quality. A more intelligible metric of how well each model fits to the experimental data is the relative likelihood, which can be derived from the BIC by using the formula:

$$Relative\ Likelihood = Exp\left[\frac{BIC(model)_{Min} - BIC(model)}{2}\right] \qquad (Eq. S6)$$

where $BIC(model)_{Min}$ is the smallest BIC value from the model fits. By definition, the model whose fit minimizes the BIC has a Relative Likelihood of 1 while all other models have Relative Likelihood <1, making it the most descriptive diffusion model from the array of plausible models defined above.

To enhance the robustness of this approach, we have introduced a second goodness-of-fit metric. We ensure that all free parameters in a given model fit converged to non-zero value with a significance level of $p<0.05$. The t-statistic values are determined as the fit parameter estimates divided by the standard errors, and the p-values reported are the two-sided p-values for the t-statistic, with the null hypothesis being that the parameter is equal to zero. This is introduced given that the parameters considered, especially the Diffusion coefficients, tend to assume very low values. Using these two metrics, the most likely model to describe each trajectory therefore has to satisfy two main conditions: minimizing the BIC (i.e., the highest Relative Likelihood), and that the fit parameters are significantly different than zero. If the most likely fit does not satisfy the second condition, the model with the second-smallest BIC, but which satisfies the t-test condition is then selected, and so on. Finally, if no model is able to simultaneously satisfy both conditions, then the trajectory is excluded from further analysis. Additionally, in the case of single trajectory analysis, which naturally suffers from greater noise than the ensemble average of all the curves, we also require that the coefficient of variation (R^2) for each diffusion model fit has to satisfy $R^2>0.9$ for the trajectory to be included in the analysis. This modified data analysis approach establishes a robust unbiased statistical methodology for determining the most likely type of diffusive motion model, from a particular given set of evaluated motion models, however our approach cannot provide judgement on non-tested models.

DETERMINATION OF LOCALIZATION PRECISION

A key aspect of our data analysis approach is that we have directly included the dynamic localization uncertainty, δ_{xy}, as a free parameter in the least squares fit. This is a different approach to the majority of the model fitting routines presented in relevant literature, where this quantity is either not taken into account during the derivation of parameters relevant to describe the diffusive motion of the particles, or just used as a baseline value to subtract to the entire dataset [40]. This is a crucial point, as it is evident from Eq. S4 that the localization uncertainty, especially for kHz or faster sampling rates, even slight under-estimates by a few nanometers of the localization precision can lead to inflated diffusion coefficients at short time intervals [22, 27]. Such subtle differences could for example originate from that the effect of the localization precision is determined by measurements of the static localization precision with immobile probe particles, rather than with measurements of the dynamic localization precision from mobile particles [27, 41]. The static and dynamic localization precision as determined by our ensemble average analysis approach for the time interval $0.5 \le t \le 50$ ms is shown in Table S2. This result confirms that for our ISCAT data the dynamic localization precision, as determined from analysis of mobile gold particles, is greater than the static localization precision, as determined from analysis of immobile particles.

Table S2. Static and dynamic localization precision of ISCAT microscope.

	Data Set	Most Likely Model	δ_{xy} (nm)
Static Localization Precision [1]	Ø20nm Gold / Glass (N=32)	Immobile (Eq. 7, Table 2)	9.3±0.1
	Ø40nm Gold / Glass (N=31)	Free diffusion (Eq. 2, Table 2)	8.1±0.1
Dynamic Localization Precision [2]	Ø20nm Gold / DSPE / PtK2 cells (N=229)	Transient Compartmentalized diffusion (Eq. 5.2, Table 2)	14.2±0.2
	Ø40nm Gold / DSPE / PtK2 cells (N=433)	Transient Compartmentalized diffusion (Eq. 5.2, Table 2)	13.5±0.2

[1] Localization precision, δ_{xy}, determined from immobilized gold particles on glass from fitting the time-dependence of the ensemble average apparent diffusion coefficient $D_{app}(t)$ for the time interval 0.5 ≤ t ≤ 50 ms with Eq. 1 as described in the main text and Supplementary Information. This measure for immobilized particles represents the static localization precision and is the ultimate performance limit of our ISCAT microscope at our sampling acquisition rate of 2 kHz.
[2] Localization precision, δ_{xy}, determined from mobile gold particles on Ptk2 cells from fitting the time-dependence of the ensemble average apparent diffusion coefficient $D_{app}(t)$ for the time interval 0.5 ≤ t ≤ 50 ms with Eq. 1 as described in the main text and Supplementary Information. This measure for mobile particles represents the dynamic localization precision at our sampling acquisition rate of 2 kHz.

MONTE CARLO SIMULATIONS OF 2D TRANSIENT COMPARTMENTALIZED DIFFUSION IN A HETEROGENEOUS LATTICE

To validate our experimental data analysis approach and results, we also analysed simulated trajectories for diffusion in a heterogeneous lattice, generated through the Voronoi tessellation algorithm, with a characteristic average compartment size, L_S, *as previously described* [3]. In brief, in these simulations, we generated fluorescence time traces of 2-dimensional diffusion of single molecules in a heterogeneous corralled environment. The corrals are randomly generated via a Voronoi tessellation algorithm, with randomly selected seeds, to simulate the heterogeneity of the cellular membrane environment. The simulation area was a square with side lengths of 8 to 20 µm (the dimensions of the area are not influential) and the compartmentalisation of this area was implemented as a Voronoi mesh on a uniform random distribution of seed points. We defined the square root of the average compartment area as the average compartment size or length L. The average compartment size (L), defined as the square root of the average compartment area, the hopping probability (P_{hop}) and the free diffusion coefficient (D_S) completely described our simulation model. Within a compartment the molecules were assumed to diffuse freely while crossing from one compartment to another is regulated by a "hopping probability" P_{hop}. This was implemented in the following way: if the diffusion motion (with diffusion coefficient D_S) would make the lipid to cross the compartment boundary, a random number is generated, and the movement takes place only if this number is above the threshold defined by P_{hop}. In all other cases, a new displacement is calculated, where the molecule would be diffusing in the same compartment. In the special case of free Brownian diffusion (i.e. P_{hop} =1) each collision with a compartment boundary results in a molecule crossing to the adjacent compartment. When P_{hop}<1, e.g. for P_{hop}=1/40, only 1 out of 40 collisions with a compartment boundary results in a molecule crossing to the adjacent compartment. For each condition (D_S, L_S, and P_{hop}), we simulated N=100 trajectories with 0.5 ms time steps (i.e. a sampling rate of 2 kHz) and a time span of 250 ms (i.e. 500 displacements per trajectory). To better approximate experimental conditions, we further add a simulated localization precision term $\delta_{xy}{}^S$ to each individual localization in a simulated trajectory, in place of the experimental localization precision δ_{xy} that would exist for experimental data. The simulated localization precision in this instance was done by adding a random number from a normal distribution with a mean of zero and variance of 0.5 $(\delta_{xy}{}^S)^2$,N[0,0.5$(\delta_{xy}{}^S)^2$] to each simulated trajectory co-ordinate position along the x-axis, and a second random number from the same distribution along the y-axis, where numbers were added in a random direction along the x-axis, and a re-generated random direction along the y-axis. This ensures that the distribution for the added localization precision in the xy-plane is given by a normal distribution N[0, $(\delta_{xy}{}^S)^2$]. Simulated trajectories were subsequently analysed by use of same data analysis pipeline as for the experimental ISCAT data, except that the camera blur correction factor was set to R=0.

In order to attempt to verify the accuracy of our ensemble average analysis workflow, we simulated sets of 100 trajectories with trajectory lengths and sampling frequency that were equivalent to our experimental data sets of 500 localizations, and sampling frequency of 2 kHz. In these simulations, we first explored the parameter space of the simulation parameters D_S, L_S, and P_{Hop}, plus the addition of simulated localization precision as described above with an aim to find simulated conditions that matched the ensemble average time-dependence of our experimental data for lipids labelled with either Ø20 nm, or Ø40 nm gold particles as shown in Figure 1d. While there are no absolute predictions for the relationship between the simulation parameters and the shape of the time-dependence of the apparent diffusion coefficient $D_{app}(t)$, a few generalized observations are: 1) The magnitude of the apparent diffusion coefficient $D_{app}(t)$ at intermediate times of ≈2 ≤ n t_{lag} ≤ ≈ 10 ms depends primarily on the simulated intra-compartmental diffusion coefficient D_S but with the added effect of the artificial diffusion-like term that depends on the localization precision in the limit of smaller times n t_{lag} < ≈ 2ms. 2) The magnitude of the apparent diffusion coefficient $D_{app}(t)$ at longer times of n t_{lag} ≥ ≈ 10 ms depends primarily on the simulated hopping probability P_{Hop}. 3) The transition time from faster to slower diffusion depends primarily on the confinement size L_S. With this in mind, it is possible to systematically explore the parameter space with gradually adjusted simulation parameters in order to obtain matching conditions to the experimental data. In the case of this work, we found that the experimental data for the diffusion of lipids labelled with the smaller Ø20 nm gold particles could be well matched by simulated trajectories with simulation parameters of P_{Hop} = 0.06, D_S = 1.1 µm^2/s, L_S = 120nm, and $\delta_{xy}{}^S$ = 16 nm. In the case of this work, we found that the experimental data for the diffusion of lipids labelled with the smaller Ø20 nm gold particles could be well matched by simulated trajectories with simulation parameters of P_{Hop} = 0.04, D_S = 0.8 µm^2/s, L_S = 120nm, and $\delta_{xy}{}^S$ = 16 nm. The overlap of the ensemble average time-dependence of the apparent diffusion coefficient D_{app}(n t_{lag}) between simulations with these parameters and our experimental data is shown in Figure 4. Using these simulated trajectories, we were then able to explore the suitability and accuracy of our quantitative ensemble average and single trajectory analysis pipelines to the simulated trajectories at analysis time ranges 0.5 ≤ t ≤ T with T=5ms, 10ms, 25ms, 50ms, 75ms, and

100ms. The results of the ensemble average analysis are shown in the main text, with additional details in Supplemental Tables S7 and S8, and Supplemental Figure S4.

We further used the simulations as a means to compare experimental data from different methods (Figure 5). For this we simulated 100 trajectories with trajectory lengths and sampling frequency that were equivalent to our experimental data sets of 500 localizations, and sampling frequency of 2 kHz and with simulation parameters D_S=1.0 µm^2/s, L_S=120 nm, δ_{xy}^S=20 nm, and P_{Hop} =1, 0.5, 0.25, 0.1, 0.05, and 0.025, where the choice of the magnitude of the simulation parameters were selected to roughly match recent results for a broad range of experimental data from ISCAT, SPT, STED-FCS, FCS, and SFMI. To generate a trend line we subsequently fit the resulting S_{Conf} versus P_{Hop} points to a fourth order polynomial. This trend line was then used to estimate the P_{Hop} parameter for experimental SPT, FCS, and SFMI data.

Supplementary Videos

Supplementary Video 1. Continuous ISCAT frames of a phospholipid labeled with the scattering Ø20 nm gold particle moving in the plasma membrane of a live PtK2 cell. The intensity of the ISCAT images was inverted for the video to let the scattering particle appear as bright spot with a dark peripheral background. The dark periphery shows as an artefact, since we subtracted the average signal over the whole image for each video frame, and since the particle spends many frames (due to the high framerate) almost in one place. Scale bar 1 µm.

Supplementary Video 2. Continuous ISCAT frames of a phospholipid labeled with the scattering Ø20 nm gold particle moving in the plasma membrane of a live PtK2 cell. The intensity of the ISCAT images was inverted for the video to let the scattering particle appear as bright spot with a dark peripheral background. The dark periphery shows as an artefact, since we subtracted the average signal over the whole image for each video frame, and since the particle spends many frames (due to the high framerate) almost in one place. Scale bar 1 µm.

Supplemental Table S3. Parameters for the model fit to the ensemble average curve of the Apparent Diffusion Coefficient data for all trajectories of Ø20nm diameter gold nanoparticles-tagged DSPE lipids on PtK2 cells

Model [1]	BIC	Relative Likelihood	D_μ (±S.E.) (μm²/s)	D_M (±S.E.) (μm²/s)	L (±S.E.) (nm)	τ_{Conf} (ms)	δ_{xy} (±S.E.) (nm)	S_{Conf} (±std)
Analysis Time Range: 0.5 ≤ t ≤ 5 ms								
Compartmentalized Model 1 (Approximate)	-129.9	0.72	0.88±0.003	0.42±0.001	99±0.4	5.8±0.1	13.6±0.1	2.1±0.01
Compartmentalized Model 2 (Exact)	-130.5	1.00	0.98±0.004	0.42±0.001	95±0.3	5.3±0.1	13.4±0.1	2.3±0.01
Analysis Time Range: 0.5 ≤ t≤ 10 ms								
Compartmentalized Model 1 (Approximate)	-197.8	0.92	0.87±0.009	0.42±0.001	100±0.8	5.9±0.1	13.6±0.1	2.1±0.02
Compartmentalized Model 2 (Exact)	-198.0	1.00	0.97±0.011	0.42±0.001	96±0.7	5.5±0.1	13.5±0.1	2.3±0.03
Analysis Time Range: 0.5 ≤ t ≤ 25 ms								
Compartmentalized Model 1 (Approximate)	-241.3	0.88	0.82±0.02	0.41±0.02	110±2	7.0±0.2	14.1±0.2	2.0±0.04
Compartmentalized Model 2 (Exact)	-241.6	1.00	0.91±0.02	0.41±0.02	100 ±2	6.5±0.2	13.9±0.2	2.2±0.05
Analysis Time Range: 0.5 ≤ t ≤ 50 ms								
Compartmentalized Model 1 (Approximate)	-242.0	0.86	0.78±0.02	0.40±0.004	110±3.0	8.1±0.4	14.4±0.2	2.0±0.05
Compartmentalized Model 2 (Exact)	-242.3	1.00	0.87±0.02	0.40±0.004	110 ±3.0	7.5±0.4	14.2±0.2	2.2±0.06
Analysis Time Range: 0.5 ≤ t ≤ 75 ms								
Compartmentalized Model 1 (Approximate)	-235.9	0.84	0.77±0.02	0.39±0.005	120±4	8.8±0.6	14.5±0.2	2.0±0.06
Compartmentalized Model 2 (Exact)	-236.3	1.00	0.85±0.02	0.39±0.005	110±4	8.1±0.5	14.4±0.2	2.2±0.07
Analysis Time Range: 0.5 ≤ t ≤ 100 ms								
Compartmentalized Model 1 (Approximate)	-237.9	0.82	0.76±0.020	0.39±0.005	120±3.9	9.2±0.6	14.6±0.2	1.9±0.06
Compartmentalized Model 2 (Exact)	-238.3	1.00	0.84±0.02	0.39±0.005	120±3.8	8.5±0.6	14.4±0.2	2.2±0.07

[1] The data was fitted for all the models from Table 2 but we only report on the fit results for models for which the Relative Likelihood is larger than 0.5.The data for the most likely fit model is also plotted in Figure 2.

Supplemental Table S4. Parameters for the model fit to the ensemble average curve of the Apparent Diffusion Coefficient data for all trajectories of Ø40nm diameter gold nanoparticles-tagged DSPE lipids on PtK2 cells.

Model [1]	BIC	Relative Likelihood	D_μ (±S.E.) ($\mu m^2/s$)	D_M (±S.E.) ($\mu m^2/s$)	L (±S.E.) (nm)	τ_{Conf} (ms)	δ_{xy} (±S.E.) (nm)	S_{Conf} (±std.)
Analysis Time Range: 0.5 ≤ t ≤ 5 ms								
Compartmentalized Model 1 (Approximate)	-120.5	0.78	0.70±0.005	0.29±0.001	89±0.5	6.9±0.1	12.7±0.0	2.4±0.02
Compartmentalized Model 2 (Exact)	-121.0	1.00	0.79±0.006	0.29±0.001	86±0.5	6.5±0.1	12.6±0.0	2.8±0.02
Analysis Time Range: 0.5 ≤ t ≤ 10 ms								
Compartmentalized Model 1 (Approximate)	-189.3	0.89	0.68±0.010	0.28±0.002	91±0.9	7.4±0.2	12.9±0.1	2.4±0.04
Compartmentalized Model 2 (Exact)	-189.5	1.00	0.76±0.012	0.28±0.002	88±0.9	7.0±0.2	12.8±0.1	2.7±0.05
Analysis Time Range: 0.5 ≤ t ≤ 25 ms								
Compartmentalized Model 1 (Approximate)	-239.6	0.82	0.62±0.014	0.27±0.003	100±1.9	9.4±0.5	13.4±0.2	2.4±0.06
Compartmentalized Model 2 (Exact)	-240.0	1.00	0.70±0.017	0.27±0.003	97±1.8	8.8±0.4	13.2±0.2	2.7±0.07
Analysis Time Range: 0.5 ≤ t ≤ 50 ms								
Compartmentalized Model 1 (Approximate)	-258.3	0.76	0.60±0.014	0.26±0.003	110±2.4	11±0.6	13.7±0.2	2.3±0.06
Compartmentalized Model 2 (Exact)	-258.8	1.00	0.67±0.017	0.26±0.003	100 ±2.3	10±0.6	13.5±0.2	2.6±0.07
Analysis Time Range: 0.5 ≤ t ≤ 75 ms								
Compartmentalized Model 1 (Approximate)	-258.2	0.73	0.59±0.014	0.25±0.004	110±2.7	12±0.8	13.8±0.2	2.3±0.07
Compartmentalized Model 2 (Exact)	-258.8	1.00	0.66±0.017	0.25±0.004	100±2.6	11±0.7	13.6±0.2	2.6±0.08
Analysis Time Range: 0.5 ≤ t ≤ 100 ms								
Compartmentalized Model 1 (Approximate)	-263.6	0.71	0.58±0.014	0.25±0.004	110±2.8	12±0.8	13.8±0.2	2.3±0.07
Compartmentalized Model 2 (Exact)	-264.3	1.00	0.65±0.017	0.25±0.004	110±2.7	11±0.8	13.6±0.2	2.6±0.08

The data was fitted for all the models from Table 2 but we only report on the fit results for models for which the Relative Likelihood is larger than 0.5.The data for the most likely fit model is also plotted in Figure 2.

Supplemental Table S5. Parameters for the model fit to the ensemble average curve of the Apparent Diffusion Coefficient data for all trajectories of Ø20nm diameter gold nanoparticles immobilized on glass substrate.

Model [1]	BIC	Relative Likelihood	D_μ (±S.E.) ($\mu m^2/s$)	D_M (±S.E.) ($\mu m^2/s$)	L (±S.E.) (nm)	τ_{Conf} (±S.E.) (ms)	δ_{xy} (±S.E.) (nm)	S_{Conf} (±std)
Analysis Time Range: $0.5 \leq t \leq 5$ ms								
Localization Uncertainty	-88.8	1.00					9.3±0.1	
Analysis Time Range: $0.5 \leq t \leq 10$ ms								
Localization Uncertainty	-194.4	1.00					9.3±0.1	
Analysis Time Range: $0.5 \leq t \leq 25$ ms								
Localization Uncertainty	-328.8	1.00					9.3±0.1	
Analysis Time Range: $0.5 \leq t \leq 50$ ms								
Localization Uncertainty	-398.1	1.00					9.3±0.1	
Analysis Time Range: $0.5 \leq t \leq 75$ ms								
Localization Uncertainty	-421.4	1.00					9.3±0.1	
Analysis Time Range: $0.5 \leq t \leq 100$ ms								
Localization Uncertainty	-445.0	1.00					9.3±0.1	

[1] The data was fitted for all the models from Table 2 but we only report on the fit results for models for which the Relative Likelihood is larger than 0.5.The data for the most likely fit model is also plotted in Figure 2.

Supplemental Table S6. Parameters for the model fit to the ensemble average curve to the apparent Diffusion Coefficient data for all trajectories of Ø40nm diameter gold nanoparticles immobilized on glass substrate.

Model [1]	BIC	Relative Likelihood	D_μ (±S.E.) (μm²/s)	D_M (±S.E.) (μm²/s)	L (±S.E.) (nm)	τ_{Conf} (±S.E.) (ms)	δ_{xy} (±S.E.) (nm)	S_{Conf} (±std)
Analysis Time Range: 0.5 ≤ t ≤ 5 ms								
Free	-89.4	1.00		0.003±0.001			7.9±0.1	
Analysis Time Range: 0.5 ≤ t ≤ 10 ms								
Free	-190.1	1.00		0.001±0.001			8.0±0.1	
Analysis Time Range: 0.5 ≤ t ≤ 25 ms								
Free	-320.9	1.00		0.001±0.001			8.0±0.1	
Analysis Time Range: 0.5 ≤ t ≤ 50 ms								
Free	-387.2	1.00		0.001±0.0005			8.0±0.1	
Analysis Time Range: 0.5 ≤ t ≤ 75 ms								
Free	-409.3	1.00		0.001±0.0005			8.0±0.1	
Analysis Time Range: 0.5 ≤ t ≤ 100 ms								
Free	-431.8	1.00		0.001±0.0005			8.0±0.1	

[1] The data was fitted for all the models from Table 2 but we only report on the fit results for models for which the Relative Likelihood is larger than 0.5.The data for the most likely fit model is also plotted in Figure 2.

Supplemental Table S7. Ensemble average fit parameters for simulated trajectories matching the Ø20nm gold tagged DSPE lipids diffusing on PTK2 cell membranes.

Model [1]	BIC	Relative Likelihood	D_μ (±S.E.) (μm²/s)	D_M (±S.E.) (μm²/s)	L (±S.E.) (nm)	τ_{Conf} (ms)	δ_{xy} (±S.E.) (nm)	S_{Conf} (±S.E.)
Analysis Time Range: 0.5 ≤ t ≤ 5 ms								
Compartmentalized Model 1 (Approximate)	-109.71	0.987	0.87 ± 0.01	0.40 ± 0.002	97.4 ± 0.9	6.0 ± 0.2	13.1 ± 0.1	2.2 ± 0.03
Compartmentalized Model 2 (Exact)	-109.74	1	0.97 ± 0.01	0.40 ± 0.002	93.8 ± 0.9	5.5 ± 0.1	12.9 ± 0.1	2.5± 0.03
Analysis Time Range: 0.5 ≤ t ≤ 10 ms								
Compartmentalized Model 1 (Approximate)	-179.398	0.964	0.83 ± 0.01	0.39 ± 0.002	102 ± 1	6.8 ± 0.2	13.5 ± 0.1	2.1 ± 0.04
Compartmentalized Model 2 (Exact)	-179.471	1	0.93 ± 0.02	0.39 ± 0.002	98 ± 1	6.3 ± 0.2	13.3 ± 0.1	2.4 ± 0.04
Analysis Time Range: 0.5 ≤ t ≤ 25 ms								
Compartmentalized Model 1 (Approximate)	-249.565	0.916	0.79 ± 0.01	0.38 ± 0.002	107 ± 1	7.7 ± 0.2	13.9 ± 0.2	2.1 ± 0.04
Compartmentalized Model 2 (Exact)	-249.74	1	0.88 ± 0.02	0.38 ± 0.002	104 ± 1	7.2 ± 0.1	13.7 ± 0.2	2.4 ± 0.05
Analysis Time Range: 0.5 ≤ t ≤ 50 ms								
Compartmentalized Model 1 (Approximate)	-271.32	0.895	0.77 ± 0.01	0.37 ± 0.003	110 ± 2	8.2 ± 0.3	14.1 ± 0.2	2.1 ± 0.04
Compartmentalized Model 2 (Exact)	-271.54	1	0.86 ± 0.02	0.37 ± 0.003	106 ± 2	7.6 ± 0.3	13.8 ± 0.2	2.3 ± 0.05
Analysis Time Range: 0.5 ≤ t ≤ 75 ms								
Compartmentalized Model 1 (Approximate)	-273.365	0.889	0.77 ± 0.01	0.37 ± 0.003	111 ± 2	8.4 ± 0.3	14.1 ± 0.2	2.1 ± 0.04
Compartmentalized Model 2 (Exact)	-273.602	1	0.86 ± 0.02	0.37 ± 0.003	107 ± 2	7.7 ± 0.3	13.9 ± 0.2	2.3 ± 0.05
Analysis Time Range: 0.5 ≤ t ≤ 100 ms								
Compartmentalized Model 1 (Approximate)	-280.945	0.883	0.77 ± 0.01	0.37 ± 0.003	111 ± 2	8.4 ± 0.3	14.2 ± 0.2	2.1 ± 0.04
Compartmentalized Model 2 (Exact)	-281.192	1	0.86 ± 0.02	0.37 ± 0.003	107 ± 2	7.7 ± 0.3	13.9 ± 0.2	2.3 ± 0.05

[1] A set of 100 trajectories, 500 localizations long at 2kHz sampling rates were simulated, and their $D_{App}(t)$ curves averaged together. The resulting ensemble average curves where simulation parameters are: P_{Hop} = 0.06, D = 1.1 μm²/s, L = 120nm, δ_{xy} = 16 nm.

Supplemental Table S8. Ensemble average fit parameters for simulated trajectories matching the Ø40nm gold tagged DSPE lipids on PTK2 cell membranes.

Model [1]	BIC	Relative Likelihood	D_μ (±S.E.) (μm²/s)	D_M (±S.E.) (μm²/s)	L (±S.E.) (nm)	τ_{Conf} (ms)	δ_{xy} (±S.E.) (nm)	S_{Conf} (±S.E.)
Analysis Time Range: 0.5 ≤ t ≤ 5 ms								
Compartmentalized Model 1 (Approximate)	-116.202	1	0.68 ± 0.006	0.267 ± 0.002	93.4 ± 0.8	8.2 ± 0.3	12.5 ± 0.07	2.54 ± 0.03
Compartmentalized Model 2 (Exact)	-116.043	0.923	0.77 ± 0.007	0.267 ± 0.002	90.6 ± 0.7	7.7 ± 0.2	12.2 ± 0.07	2.9 ± 0.03
Analysis Time Range: 0.5 ≤ t ≤ 10 ms								
Compartmentalized Model 1 (Approximate)	-183.98	0.97	0.66 ± 0.01	0.261 ± 0.002	96 ± 1	8.9 ± 0.3	12.7 ± 0.1	2.5 ± 0.04
Compartmentalized Model 2 (Exact)	-184.07	1	0.75 ± 0.01	0.261 ± 0.002	93 ± 1	8.3 ± 0.3	12.4 ± 0.1	2.9 ± 0.05
Analysis Time Range: 0.5 ≤ t ≤ 25 ms								
Compartmentalized Model 1 (Approximate)	-261.46	0.88	0.63 ± 0.01	0.25 ± 0.002	101 ± 1	10.1 ± 0.4	13.0 ± 0.1	2.5 ± 0.04
Compartmentalized Model 2 (Exact)	-261.7	1	0.72 ± 0.01	0.25 ± 0.002	98 ± 1	9.5 ± 0.3	12.8 ± 0.1	2.9 ± 0.05
Analysis Time Range: 0.5 ≤ t ≤ 50 ms								
Compartmentalized Model 1 (Approximate)	-293.17	0.85	0.62 ± 0.01	0.25 ± 0.002	102 ± 1	10.5 ± 0.4	13.1 ± 0.1	2.5 ± 0.04
Compartmentalized Model 2 (Exact)	-293.5	1	0.71 ± 0.01	0.25 ± 0.002	99 ± 1	9.9 ± 0.4	12.9 ± 0.1	2.9 ± 0.05
Analysis Time Range: 0.5 ≤ t ≤ 75 ms								
Compartmentalized Model 1 (Approximate)	-295	0.85	0.62 ± 0.01	0.25 ± 0.002	103 ± 1	10.7 ± 0.4	13.1 ± 0.1	2.5 ± 0.05
Compartmentalized Model 2 (Exact)	-295.3	1	0.70 ± 0.01	0.25 ± 0.002	100 ± 1	10.1 ± 0.4	12.9 ± 0.1	2.9 ± 0.06
Analysis Time Range: 0.5 ≤ t ≤ 100 ms								
Compartmentalized Model 1 (Approximate)	-299.3	0.845	0.62 ± 0.01	0.25 ± 0.002	103 ± 1	10.9 ± 0.5	13.2 ± 0.1	2.5 ± 0.05
Compartmentalized Model 2 (Exact)	-299.63	1	0.70 ± 0.01	0.25 ± 0.002	100 ± 1	10.2 ± 0.4	12.9 ± 0.2	2.9 ± 0.06

[1] A set of 100 trajectories, 500 localizations long at 2kHz sampling rates were simulated, and their $D_{App}(t)$ curves averaged together. The resulting ensemble average curves where simulation parameters are: P_{hop} = 0.04, D = 0.8 μm²/s, L = 120nm, δ_{xy} = 16 nm)

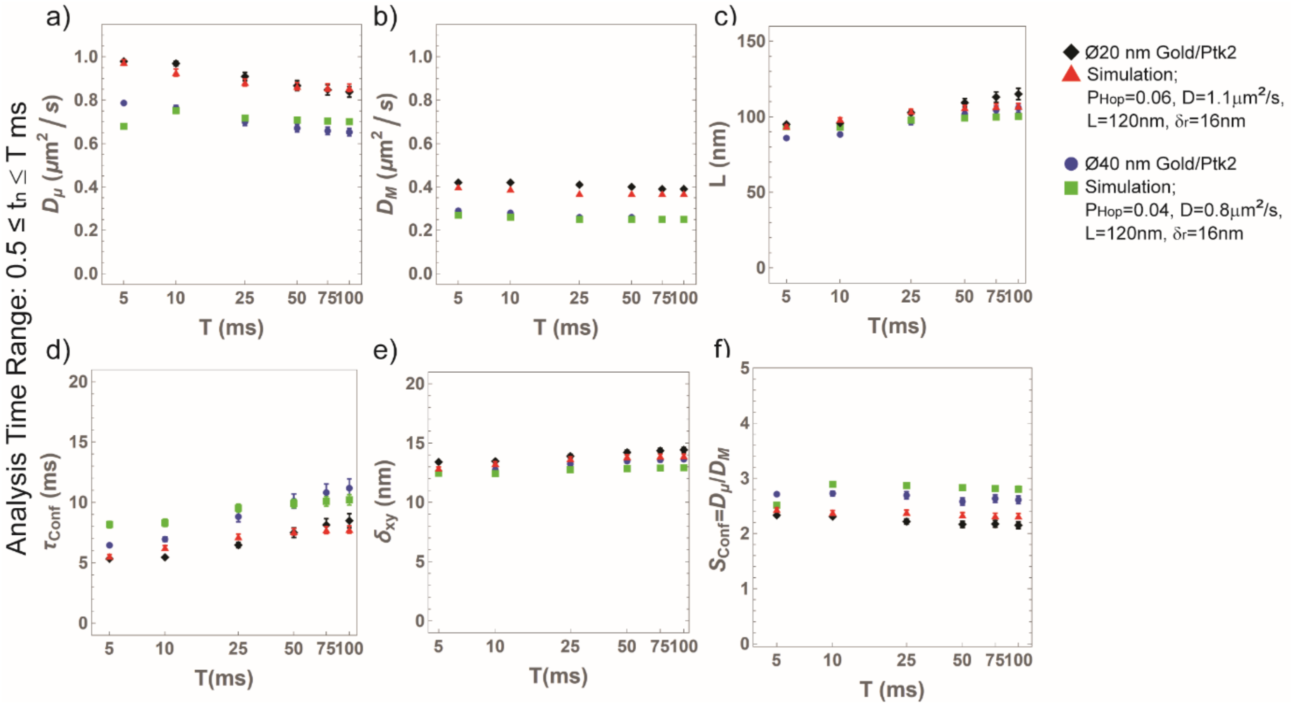

Supplemental Figure S4. Comparison between average fit parameters between experimental data and matching simulated data. a–f) The ensemble average $D_{App}(t)$ curves obtained from the trajectories of Ø20 nm gold-tagged DSPE lipids on PTK2 cell membranes (black diamonds), simulated trajectory data for compartmentalized diffusion in a heterogenous lattice with simulated parameters P_{Hop}=0.06, D_S=1.1 μm^2/s, L_S=120 nm, and δ_{xy}^S=16 nm (red triangles), Ø40 nm gold-tagged DSPE lipids on Ptk2 cell membranes (black circles), and simulated trajectory data with simulated parameters P_{Hop}=0.04, D_S=0.8 μm^2/s, L_S =120 nm, and δ_{xy}^S =16 nm (green squares) were analysed at different time intervals (0.5 ≤ t ≤ T ms with T= 5, 10, 25, 50, 75, and 100 ms). The most likely model of diffusion for all data sets and time intervals was the exact compartmentalized diffusion (Eq. 5.2) with localization uncertainty. We plot here the resulting fit parameters, obtained from the fitting the most likely diffusion model to the data, and the resulting confinement strength metrics. The values shown here shown here are: a) the unhindered intra-compartmental diffusion coefficient D_μ, b) the inter-compartmental diffusion coefficient D_M, c) the confinement size L, d) the confinement time τ_{Conf}=L^2/(4 D_M), e) the localization precision δ_{xy}, and the confinement strength S_{Conf} = D_μ / D_M.

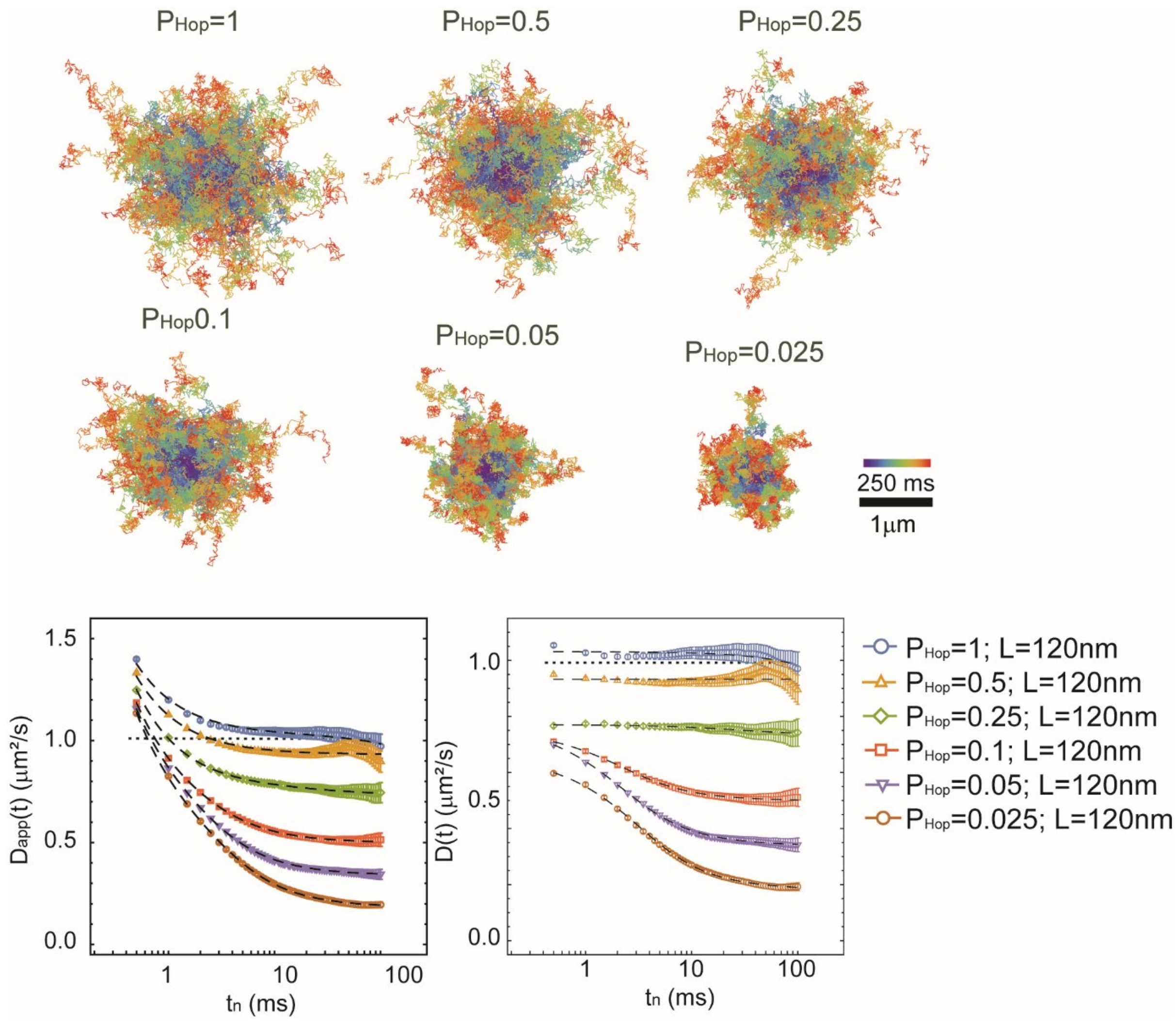

Supplemental Figure S5. Simulated single molecule tracking data. a) Superimposed simulated trajectories at sampling frequency of 2kHz (N=100 trajectories for each case) of diffusion in a heterogeneous lattice with a characteristic compartment length of L=120 nm, a free diffusion coefficient D=1.0 μm^2/s, and a localization error of δ_{xy}=20 nm, as a function of the hopping probability, P_{Hop}, ranging from as indicated in Figure from P_{Hop}=1 (i.e. free diffusion as each incidence of a molecule with a barrier results in molecule escaping to adjacent corral in the lattice) to P_{Hop}=0.025 (i.e. only 1 out 40 incidences of a molecule with a barrier results in molecule escaping to adjacent corral in the lattice while remaining 39 incidences results in retention of molecule in the same corral). All trajectories were truncated to 250 ms (n=500 displacements) long segments, re-mapped to start at the same position. The color scale indicates the time of each localization (blue to red). b) Calculated average time dependence of the apparent diffusion coefficient $D_{app}(t)=MSD_{app}(t)/(4t)$ for data in a) where dotted line corresponds to simulated diffusion coefficient $D_{Simulated}$=1.0 μm^2/s. c) Same data shown in b), but where the effect of the localization uncertainty, as determined from the fit parameters for the most likely motion model to the average $D_{app}(t)$ time dependence, has been subtracted. The fit results for the most likely models are also shown in Supplementary Table S9 for the analysis time range of $0.5 \le t \le 50$ ms.

Table S9. Ensemble Average Analysis Results of simulated trajectories of diffusion in a continuous lattice with analysis time range: 0.5 ≤ t ≤ 50 ms. Simulated parameters were D_S=1.0 μm²/s, L_S=120 nm, and $\delta_{r\,S}$=20 nm.

Model	Most Likely Model	D_μ (±S.E.) (μm²/s)	D_M (±S.E.) (μm²/s)	L (±S.E.) (nm)	δ_{xy} (±S.E.) (nm)	S_{Conf} (±s.t.d.)
P_{Hop}=1	Free	1.02±0.00	1.02±0.00		19.0±0.1	1
P_{Hop}=0.5	Free	0.93±0.00	0.93±0.00		19.7±0.3	1
P_{Hop}=0.25	Compartmentalized	0.77±0.01	0.73±0.01	98±16	22.0±0.1	1.1 ± 0.0
P_{Hop}=0.1	Compartmentalized	0.74±0.00	0.50±0.00	64±0.7	22.1±0.1	1.5 ± 0.0
P_{Hop}=0.05	Compartmentalized	0.78±0.02	0.34±0.00	81±2.0	21.3±0.3	2.3 ± 0.0
P_{Hop}=0.025	Compartmentalized	0.67±0.01	0.18±0.00	100±1.4	23.0±0.2	3.7 ± 0.0

Supplemental Table S10. Comparison of values for Diffusion coefficients, S_{conf}, τ_{Conf} and other relevant parameters obtained from the diffusion experiments and simulations from this study, with those obtained from related literature.

Method	Probe	Cell line	Analysis	Analysis Time Range	D_{Fast} (μm²/s)	D_{Slow} (μm²/s)	L (nm)	S_{Conf}	τ_{Conf} (ms)
ISCAT [1]	biotin-PEG2000-DSPE / sAv-Au (Ø20nm)	Ptk2	Ensemble Average	0.5 ≤ t ≤ 50 ms	0.87±0.02	0.40±0.00†	110±3	2.2 ± 0.1	7.5 ± 0.4
			Transient Compartmentalized Diffusion Subset (88/225 trajectories)		0.8±0.4	0.3±0.2	130±50	2.7 ± 2.2	30 ± 30
Simulation P_{Hop}=0.06; D_S=1.1 μm²/s; L_S=120 nm; δ_{xy}^S=16 nm [1]	-	-	Ensemble Average	0.5 ≤ t ≤ 50 ms	0.86±0.02	0.37±0.00†	106±2	2.3 ± 0.1	7.6 ± 0.3
ISCAT [1]	biotin-PEG2000-DSPE / sAv-Au (Ø40nm)	Ptk2	Ensemble Average	0.5 ≤ t ≤ 50 ms	0.67±0.02	0.26±0.00†	100±2	2.6 ± 0.1	10 ± 0.6
			Transient Compartmentalized Diffusion Subset (174/422 trajectories)		0.9±0.6	0.2±0.1	120±40	4.5 ± 3.8	24 ± 25
Simulation P_{Hop}=0.04; D_S=0.8 μm²/s; L_S=120 nm; δ_{xy}^S=16 nm [1]	-	-	Ensemble Average	0.5 ≤ t ≤ 50 ms	0.71±0.01	0.25±0.00	99±1	2.9±0.1	9.9 ± 0.4
STED-FCS [2]	Atto647N-DPPE	IA32 MEF (Ink4a/Arf (-/-))	Ensemble Average	≈ 0.5 ≤ t ≤ ≈ 30 ms [3]	0.80±0.03	≈ 0.4	150±12	≈ 2.0 [4]	14 ± 2
STED-FCS [2]	Atto647N-DPPE	NRK	Ensemble Average	≈ 0.5 ≤ t ≤ ≈ 30 ms [3]	0.80±0.03	≈ 0.3	80±8	≈ 2.7 [4]	5.3 ± 0.5
FCS (+/-CK-666) [5]	Atto647N-DPPE	Ptk2	Ensemble Average	≈ 0.5 ≤ t ≤ ≈ 30 ms [3]	≈ 0.70 (+CK666)	≈ 0.40 (-CK666)	N/A	≈ 1.8 [4]	N/A

STED-FCS [6]	Atto647N-DPPE	Ptk2	Ensemble Average	≈ 0.5 ≤ t ≤ ≈ 30 ms [3]	≈ 0.40	≈ 0.40	N/A	≈ 1.0 [4]	0
SPT [7]	biotin-cap-DPPE / sAv-QD655	IA32 MEF (Ink4a/Arf (-/-))	Ensemble Average	≈ 0.6 ≤ t ≤ 50 ms	0.78±0.06	0.078±0.001	110±2	10±0.8	39 ± 1
SFMI [8]	Cy3-DOPE	T24	Single trajectory Analysis	N/A	0.83±0.056	0.30±0.021	107±6.2	2.8±0.27	9.2±0.34

[1] Data featured in this work.
[2] Data featured in [3].
[3] For STED-FCS, and FCS data the analysis time range corresponds to the accesible correlation time range from ~0.5 ms at the highest STED power for lipid diffusion in model membranes, to ~30 ms for the lipid diffusion in the plasma membrane at conventional confocal FCS
[4] For STED-FCS measurements, we consider D_{Fast} the diffusion coefficient calculated from data acquired at the highest available STED power (corresponding to an approximate lateral resolution of 50 nm for the specific studies), whereas we have defined D_{Slow} as the diffusion coefficient calculated from data acquired at confocal lateral resolution (~250 nm). Thus, the confinement strength, S_{Conf}, for STED-FCS is defined as $S_{Conf} = D_{Fast}/D_{Slow} = D_{STED}/D_{Confocal}$.
[5] For conventional FCS measurements with and without Arp2/3 specific inhibitor CK-666, we have defined D_{Fast} as the diffusion coefficient calculated from measurements in the presence of 100 µM CK-666, whereas D_{Slow} is the extracted diffusion coefficient for data acquired in the absence of CK-666. Thus, the confinement strength, S_{Conf}, for FCS in this case is defined as $S_{Conf} = D_{Fast}/D_{Slow} = D_{Confocal}^{+CK666}/D_{Confocal}^{-CK666}$.
[6] STED-FCS data featured in [8].
[7] Single particle tracking data with quantum dots featured in [22].
[8] Single fluorescent-molecule imaging (SFMI) data featured in [2]
